# Survival and safety after neoadjuvant chemotherapy or upfront surgery for locally advanced colon cancer: meta-analysis

**DOI:** 10.1093/bjs/znae021

**Published:** 2024-02-21

**Authors:** Daniel Aliseda, Jorge Arredondo, Carlos Sánchez-Justicia, Alicia Alvarellos, Javier Rodríguez, Ignacio Matos, Fernando Rotellar, Jorge Baixauli, Carlos Pastor

**Affiliations:** Department of General Surgery, Division of Colorectal Surgery, Clinica Universidad de Navarra, University of Navarra, Pamplona-Madrid, Spain; Department of General Surgery, Division of Colorectal Surgery, Clinica Universidad de Navarra, University of Navarra, Pamplona-Madrid, Spain; Institute of Health Research of Navarra (IdisNA), Pamplona, Spain; Department of General Surgery, Division of Colorectal Surgery, Clinica Universidad de Navarra, University of Navarra, Pamplona-Madrid, Spain; Institute of Health Research of Navarra (IdisNA), Pamplona, Spain; Department of General Surgery, Division of Colorectal Surgery, Clinica Universidad de Navarra, University of Navarra, Pamplona-Madrid, Spain; Institute of Health Research of Navarra (IdisNA), Pamplona, Spain; Department of Oncology, Clinica Universidad de Navarra, University of Navarra, Pamplona-Madrid, Spain; Institute of Health Research of Navarra (IdisNA), Pamplona, Spain; Department of Oncology, Clinica Universidad de Navarra, University of Navarra, Pamplona-Madrid, Spain; Department of General Surgery, Division of Colorectal Surgery, Clinica Universidad de Navarra, University of Navarra, Pamplona-Madrid, Spain; Institute of Health Research of Navarra (IdisNA), Pamplona, Spain; Department of General Surgery, Division of Colorectal Surgery, Clinica Universidad de Navarra, University of Navarra, Pamplona-Madrid, Spain; Institute of Health Research of Navarra (IdisNA), Pamplona, Spain; Department of General Surgery, Division of Colorectal Surgery, Clinica Universidad de Navarra, University of Navarra, Pamplona-Madrid, Spain; Institute of Health Research of Navarra (IdisNA), Pamplona, Spain

## Abstract

**Background:**

Neoadjuvant chemotherapy is increasingly used to treat locally advanced (T3–4 Nx–2 M0) colon cancer due to its potential advantages over the standard approach of upfront surgery. The primary objective of this systematic review and meta-analysis was to analyse data from comparative studies to assess the impact of neoadjuvant chemotherapy on oncological outcomes.

**Methods:**

A systematic review was conducted by searching the MEDLINE and Scopus databases. The search encompassed RCTs, propensity score-matched studies, and controlled prospective studies published up to 1 April 2023. As a primary objective, overall survival and disease-free survival were compared. As a secondary objective, perioperative morbidity, mortality, and complete resection were compared using the DerSimonian and Laird models.

**Results:**

A total of seven studies comprising a total of 2120 patients were included. Neoadjuvant chemotherapy was associated with a reduction in the hazard of recurrence (HR 0.73, 95% c.i. 0.59 to 0.90; *P* = 0.003) and death (HR 0.67, 95% c.i. 0.54 to 0.83; *P* < 0.001) compared with upfront surgery. Additionally, neoadjuvant chemotherapy was significantly associated with higher 5-year overall survival (79.9% *versus* 72.6%; *P* < 0.001) and disease-free survival (73.1% *versus* 64.5%; *P* = 0.028) rates. No significant differences were observed in perioperative mortality (OR 0.97, 95% c.i. 0.28 to 3.33), overall complications (OR 0.95, 95% c.i. 0.77 to 1.16), or anastomotic leakage/intra-abdominal abscess (OR 0.88, 95% c.i. 0.60 to 1.29). However, neoadjuvant chemotherapy was associated with a lower risk of incomplete resection (OR 0.70, 95% c.i. 0.49 to 0.99).

**Conclusion:**

Neoadjuvant chemotherapy is associated with a reduced hazard of recurrence and death, as well as improved overall survival and disease-free survival rates, compared with upfront surgery in patients with locally advanced colon cancer.

## Teaser

This systematic review and meta-analysis of seven studies involving 2120 patients with locally advanced colon cancer found that neoadjuvant chemotherapy is associated with a reduced hazard of recurrence and death, as well as improved 5-year overall survival and disease-free survival rates, compared with upfront surgery. No significant differences in perioperative complications or mortality were found, but neoadjuvant chemotherapy decreased the risk of incomplete resection. In conclusion, neoadjuvant chemotherapy appears to offer oncological benefits in the treatment of locally advanced colon cancer.

## Introduction

Colon cancer (CC) is the fourth most common cancer worldwide, with approximately 1.2 million new diagnoses in 2020. In the same year, approximately 577 000 patients died from CC, with this number projected to double by 2040^1^. The recommended treatment for locally advanced disease, defined by extramural or nodal invasion, includes tumour resection followed by adjuvant chemotherapy based on fluoropyrimidines and oxaliplatin for patients with stage III disease or stage II disease with multiple high-risk factors^2^. Preoperative systemic treatment has demonstrated efficacy in the treatment of certain gastrointestinal tumours, including pancreatic, rectal, and gastric cancer, and is even being considered for the treatment of some initially resectable tumours^3–5^.

Neoadjuvant chemotherapy (NAC) has been proposed as a safe and effective therapeutic option for locally advanced colon cancer (LACC), offering several advantages over the recommended adjuvant protocol^6^. According to current clinical guidelines, NAC is recommended as an alternative treatment option specifically for individuals with cT4 CC. This recommendation is considered weak due to the reliance on moderate-quality evidence demonstrating improvements in survival outcomes and rates of clear resection margins associated with NAC in this patient population. Therefore, NAC is considered a viable treatment option for patients with these disease-specific characteristics^2,4,7^. Although NAC has demonstrated safety in terms of toxicity, and efficacy with respect to a reduction in tumour size and pathological response, its impact on long-term survival has yet to be definitively established. Accordingly, NAC is not currently considered the gold standard treatment for this subset of patients. Furthermore, the administration of NAC before surgery may increase the risk of perioperative complications, which can delay or preclude adjuvant treatment^8^. In addition, accurately assessing tumour status through radiological methods remains challenging in LACC and can result in overtreatment due to overstaging^9^.

Considering these factors, a systematic review and meta-analysis was conducted using reconstructed data from individual participants in propensity score-matched studies, prospective controlled studies, and RCTs. The aim of the present study was to provide a more reliable and precise analysis of the impact of NAC on oncological outcomes and perioperative morbidity.

## Methods

### Search strategy, study selection, and data extraction

A comprehensive search was conducted using the MEDLINE (via Ovid) and Scopus databases covering the interval from the inception of the databases up to 1 April 2023. The search utilized relevant keywords and Medical Subject Headings (MeSH) terms related to ‘locally advanced cancer stage’, ‘colon cancer’, and ‘neoadjuvant therapy’. The detailed search strategy is provided in *Appendix S1*. To ensure a thorough search, the reference lists of articles and reviews were manually screened. All eligible studies published in peer-reviewed journals and written in English were included in the study analysis. Covidence software (www.covidence.org) was used to manage uploaded studies. Two authors (D.A. and C.S.-J.) independently conducted the article selection process, initially screening titles and abstracts followed by a full-text assessment for eligibility and inclusion. Any articles that did not meet the inclusion criteria were appropriately identified and excluded. In cases where discrepancies arose during the selection process, a third author (J.A.) was consulted and consensus was reached. Relevant information, including study characteristics, patient demographics, tumour characteristics, and details of neoadjuvant and adjuvant treatments, was extracted from each included article by two independent authors using a customized form specifically created for this study.

### Eligibility criteria, objectives, and outcomes

RCTs, prospective controlled studies, and propensity score-matched studies comparing one arm of patients diagnosed with potentially resectable and non-metastatic LACC treated with NAC *versus* at least one arm of patients treated with upfront surgery and adjuvant therapy were eligible. The inclusion criteria for the studies were as follows: biopsy-confirmed LACC (T3–4 Nx–2 M0); neoadjuvant and adjuvant therapy using chemotherapy alone. Studies that included rectal cancer, included early-stage (T1 or T2) CC, included recurrent tumours, or focused on radiation therapy or chemoradiation as neoadjuvant or adjuvant treatment were excluded, as were retrospective cohort or case–control studies, reviews, editorials, case reports, meta-analyses, and conference abstracts. Patients presenting with bowel obstruction were also included in this study. The primary objective was to compare overall survival (OS) (considered as months from diagnosis to death, regardless of disease recurrence) and disease-free survival (DFS) (considered as months after diagnosis with no signs or symptoms of recurrence). As a secondary objective, postoperative outcomes (overall morbidity according to Clavien–Dindo^10^, perioperative mortality up to 90 days, anastomotic leakage or intra-abdominal abscess, and rate of complete resection (R0)) were compared. The inclusion criteria for the survival analysis were studies that provided Kaplan–Meier curves detailing the OS and DFS of the entire participant group. This review was registered in PROSPERO, the international prospective register of systematic reviews (registration number CRD42023422028) (*Appendix S2*) and is reported in adherence to the PRISMA guidelines (*Appendix S3*), as well as the recommendations of Cochrane^11,12^.

### Risk-of-bias assessment

The risk of bias for the selected RCTs was assessed using Version 2 of the Cochrane risk-of-bias tool (RoB 2), whereas the risk of bias for the remaining studies was evaluated using the Newcastle–Ottawa scale (NOS)^13,14^. Two reviewers (D.A. and C.S.-J.) independently conducted evaluations in duplicate and any disagreements were resolved through consensus. The risk-of-bias ratings were presented using the robvis tool as ‘traffic-light’ plots^15^.

### Statistical analysis

All analyses were performed using STATA Version 16 (StataCorp, College Station, TX, USA). *P* values <0.050 were considered statistically significant. ORs with 95% confidence intervals were used for dichotomous variables. Statistical heterogeneity was assessed using the Cochrane Q test and *I*^2^ statistic, which quantifies the proportion of total variability across studies due to heterogeneity. *I*^2^ values of 25%, 50%, and 75% were considered to indicate low, moderate, and high heterogeneity respectively^16^. Regardless of heterogeneity, the random-effects DerSimonian and Laird model was used to pool results, as it provides more robust effect estimates than fixed-effect models. Publication bias was assessed visually using funnel plots.

#### Survival analysis

Survival data were reconstructed from published Kaplan–Meier plots using DigitizeIt software. The *ipdfc* command in STATA was used for the reconstruction process, which includes the iterative algorithm described by Guyot *et al*.^17^. Additionally, to ensure monotonicity, the pool-adjacent-violators algorithm was implemented for isotonic regression with adjacent violators replaced with their mean. In this way, patient-level survival data were transformed to estimate time-to-event parameters, while securing the monotonicity constraint^17–19^. For each study, the survival data underwent a thorough review, which included comparison of the number of at-risk patients, 1–5-year OS rates, log rank values, and HRs when available with the original data. The Kaplan–Meier estimator was used to estimate the survival function and the log rank test was used to compare the unadjusted OS data. A one-stage meta-analysis was performed using Cox proportional hazards models, with the marginal Cox regression model selected for the primary analysis, as it assumes a common baseline hazard function across studies. Stratified and shared-frailty Cox regression models were used to model for differences in baseline hazard functions across studies and between-study heterogeneity respectively^20^. To check the proportionality assumption, visual plots of predicted *versus* observed survival functions and scaled Schoenfeld residuals were used and the Grambsch–Therneau test was conducted^21^. As a non-parametric method, the difference in the restricted median survival time (RMST) was estimated using the naive Kaplan–Meier method^22^. As part of a sensitivity analysis, summary HRs were calculated for each individual study using reconstructed individual patient data sets and then combined using a conventional two-step meta-analysis. A subgroup analysis was also conducted based on study design, distinguishing between RCTs and non-RCTs.

## Results

**Fig. 1:**
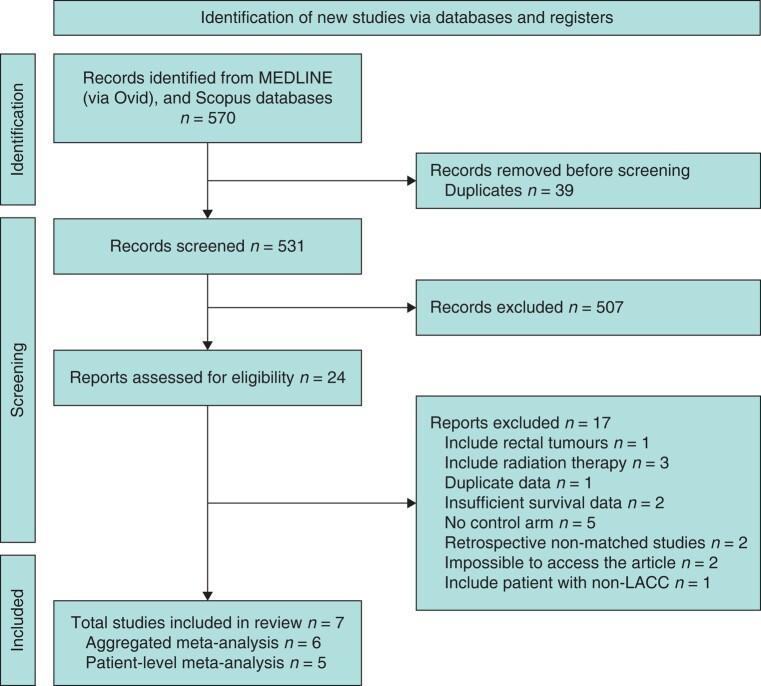
PRISMA flow chart illustrating the study selection process LACC, locally advanced colon cancer.

A systematic literature search initially yielded 570 articles, becoming 24 through title and abstract screening. A total of three studies were inaccessible; attempts were made to obtain relevant data from the corresponding author of one article, but no response was received. After reviewing the full texts of these articles, seven studies^23–29^ ultimately met the final inclusion criteria. Out of these, five studies^23,24,26,28,29^ were included in the analysis for OS, whereas six studies^23–25,27–29^ were included for assessing postoperative outcomes (*Fig. 1*). The articles that did not meet the selection criteria and reasons for their rejection are detailed in *Table S1*.

### Study and baseline patient characteristics

The analysis comprised three RCTs^26,27,29^, three propensity score-matched studies, and one prospective controlled study conducted in both Eastern and Western countries. A total of 2120 patients (diagnosed between 2008 and December 2019) were included in the analysis, with 1135 in the upfront surgery arm and 985 in the NAC arm. *Table 1* provides an overview of the study characteristics, including patient demographics, tumour characteristics, and details of the chemotherapy used. *Tables S2*, *S3* present the scores for each of the eight domains in the NOS and the five domains in the Cochrane risk-of-bias tool (RoB 2) respectively. A total of five studies received an NOS score of greater than or equal to 7 stars, indicating high methodological quality. Both RCTs were considered to have a low risk of bias.

**Table 1:** Overview of studies and patient/tumour characteristics

Study (year)	Design and interval of the study	Arm and follow-up	Number of patients	Patient characteristics	Preoperative tumour characteristics	Pathological characteristics	Chemotherapy treatment
Morton *et al*.^29^ (2023)	RCT (May 2008–Dec 2016)	NAC (FU 3.1 years; i.q.r. 2.5–4.9)	699 (686)	Mean age: 63.0 y.o (SD 9.8); male (%): 448 (64.0)	Right sided: 340 (48.7%); T4-Rx: 177 (25.4%); N1-Rx: 336 (48.1%); N2-Rx: 189 (27.1%); RAS wild type: 302 (43.3%)	pT4: 142 (20.7%); pN1: 173 (25.3%); pN2: 104 (15.2%); complete regression: 24 (3.6%)	OxFU: oxaliplatin 85 mg/m^2^ plus l-folinic acid 175 mg 2-h infusion, fluorouracil 400 mg/m^2^ bolus, 2400 mg/m^2^ 46-h infusion, repeated once every 2 weeks; regimen: 3 NAC cycles + 9 (or 3) AC or 12 (or 6) AC OxCap: oxaliplatin 130 mg/m^2^ 1-h intravenous infusion day 1, then oral capecitabine 1000 mg/m^2^ twice a day on days 1–14, repeated 3-weekly; regimen: 2 NAC cycles + 6 (or, optionally, 2) AC or 8 (or 4) AC
		Upfront surgery (FU 3.1 years; i.q.r. 2.5–4.9)	354 (351)	Mean age 63.2 y.o (SD 9.4); male (%): 225 (63.6)	Right sided: 174 (49.2%); T4-Rx: 91 (25.7%); N1-Rx: 169 (47.7%); N2-Rx: 98 (27.7%); RAS wild type: 149 (42.1%)	pT4: 107 (30.5%); pN1: 87 (25.1%): pN2: 90 (25.9%); complete regression: 0 (0%)	OxFU: 9 cycles (or 3) AC or 12 (or 6) AC OxCap: 6 cycles (or, optionally, 2) AC or 8 (or 4) AC
Zeng *et al*.^24^ (2022)	PSM study (2012–2015)	NAC (FU 56 months; range 12–80)	42	Mean age: 66.48 y.o (SD 11.98); male (%): 22 (52.4); mean BMI (kg/m^2^): 20.64 (4.37)	Right sided: 12 (28.5%); cT4: 23 (54.8%); cN2: 9 (21.4%); Median CEA ng/ml (i.q.r.): 5.6 (2.8–13.6)	pT4: 7 (16.7%); pN1: 10 (23.8%); pN2: 10 (23.8%)	6 cycles of XELOX (capecitabine 1000 mg/m^2^ orally days 1–14, oxaliplatin 130 mg/m^2^ intravenously day 1) as NAC + 2 more cycles of XELOX (based on the pathological results and the patient’s status)
		Upfront surgery (FU 66.5 months; range 2–83)	84	Mean age: 65.51 y.o (SD 11.95); male (%): 37 (44.0); mean BMI (kg/m^2^): 21.85 (4.49)	Right sided: 22 (26.2%); cT4: 57 (67.9%); cN2: 15 (17.9%); Median CEA ng/ml (i.q.r.): 4.8 (2.0–12.7)	pT4: 55 (65.5%); pN1: 24 (28.6%); pN2: 11 (13.1%)	8 cycles of XELOX (based on the pathological results and stage)
Laursen *et al*.^25^ (2022)	PSM study (1 Jan 2015– Dec 2019)	NAC	145	Median age (i.q.r.): 67 y.o (60–73); male (%): 79 (54.5); median BMI (kg/m^2^) (i.q.r.): 24.8 (22.8–28.0)	cT4: 114 (78.6%); cN2: 85 (58.6%); clinical UICC stage III: 129 (89.0%)	pT4: 51 (37.5%); pN2: 24 (17.4%); pathological UICC stage III: 63 (46.3%); adenocarcinoma: 86 (61.4%); proficient MMR: 80 (63.5%)	NR
		Upfront surgery	145	Median age (i.q.r.): 69 y.o (56–75); male (%): 85 (58.6); median BMI (kg/m^2^) (i.q.r.): 25.2 (22.8–27.6)	cT4: 111 (76.6%); cN2: 90 (62.1%); clinical UICC stage III: 131 (90.3%)	pT4: 53 (36.6%); pN2: 46 (31.7%); pathological UICC stage III: 77 (53.1%); adenocarcinoma: 110 (75.9%); proficient MMR: 100 (70.9%)	NR
Han *et al*.^30^ (2022)	Prospective controlled study (Dec 2015–Dec 2019)	NAC (FU 40 months; range 18–54)	48	Mean age: 64.3 y.o (SD: 7.2); male (%): 34 (70.8); BMI (kg/m^2^): 23.7 (2.0); albumin: (g/dl): 34.2 (2.4)	NR	pT4: 20 (41.7%); pN2: 8 (16.7%); lymphovascular invasion: 29 (60.4%)	2 cycles of CAPOX or 3 cycles of mFOLFOX6 as NAC + 6 cycles of CAPOX or 9 cycles of mFOLFOX6 as AC
		Upfront surgery (FU 35.5 months; range 10–54)	52	Mean age: 64.9 y.o (SD: 8.8); male (%): 31 (59.6); BMI (kg/m^2^): 23.1 (2.4); albumin: (g/dl): 31.5 (3.3)	NR	pT4: 27 (51.9%); pN2: 12 (23.1%); lymphovascular invasion: 29 (55.8%)	6 cycles of CAPOX or 9 cycles of mFOLFOX6 as AC
Karoui *et al*.^26^ (2021)	RCT (May 2012–May 2016)	NAC (FU 56.90 months; 95% c.i. 44.94,60.42)	52	Median age: 65 y.o (range 46–79); male (%): 30 (58); BMI (kg/m^2^) (range): 24 (18–42)	Right sided: 27 (52%); cT4: 9 (17%); cN2: 6 (12%)	pT4: 11 (23%); pN2: 7 (13%); TNM stage III: 22 (44%); tumour perforation: 2 (4%)	12 cycles (4 before surgery and 8 after surgery) of FOLFOX-4 regimen was administered intravenously for 48 h once every 2 weeks (+ cetuximab for wild-type RAS)
		Upfront surgery (FU 50.53 months, 95% c.i. 48.10,56.97)	52	Median age: 62 y.o (range 30–75); male (%): 33 (63); BMI (kg/m^2^) (range): 26 (17–39)	Right sided: 21 (40%); cT4: 3 (6%); cN2: 8 (15%)	pT4: 14 (27.5%); pN2: 16 (31%); TNM stage III: 31 (61%); tumour perforation: 3 (6%)	Only patients with stage III CC received 12 cycles of adjuvant FOLFOX-4; for those with stage II cancer, the decision to administer adjuvant chemotherapy in this arm was taken at the investigator’s discretion
Karoui *et al*.^27^ (2020)	RCT (May 2012–May 2016)	NAC	52	Median age: 65 y.o (range 46–79); male (%): 30 (58); BMI (kg/m^2^) (range): 24 (18–42)	Right sided: 27 (52%); cT4: 9 (17%); cN2: 6 (12%)	pT4: 11 (23%); pN2: 7 (13%); TNM stage III: 22 (44%); tumour perforation: 2 (4%)	12 cycles (4 before surgery and 8 after surgery) of FOLFOX-4 regimen was administered intravenously for 48 h once every 2 weeks (+ cetuximab for wild-type RAS)
		Upfront surgery	52	Median age: 62 y.o (range 30–75); male (%): 33 (63); BMI (kg/m^2^) (range): 26 (17–39)	Right sided: 21 (40%); cT4: 3 (6%); cN2: 8 (15%)	pT4: 14 (27.5%); pN2: 16 (31%); TNM stage III: 31 (61%); tumour perforation: 3 (6%)	Only patients with stage III CC received 12 cycles of adjuvant FOLFOX-4; for those with stage II cancer, the decision to administer adjuvant chemotherapy in this arm was taken at the investigator’s discretion
Gooyer *et al*.^28^ (2020)	PSM (2008–2016)	NAC (FU 44 months; range 4–133)	149	Median age: 66 y.o; male (%): 74 (49.7)	Caecum: 28 (18.8%); cT4b: 112 (75.2%); cN2: 18 (12.1%)	pT4: 84 (56.4%); pN2: 18 (12.1%); mean number of positive lymph nodes (95% c.i.): 1.3 (0.8,1.8)	NR
		Upfront surgery (FU 44 months; range 0–133)	298	Median age: 66 y.o; male (%): 155 (52.0)	Caecum: 63 (21.1%); cT4b: 221 (74.2%); cN2: 51 (17.1%)	pT4: 192 (64.4%); pN2: 107 (35.9%); mean number of positive lymph nodes (95% c.i.): 3.6 (3.1,4.1)	NR

NAC, neoadjuvant chemotherapy; FU: follow-up; i.q.r., interquartile range; AC, adjuvant chemotherapy; PSM, propensity score-matched; CEA, carcinoembryonic antigen; UICC, Union for International Cancer Control; SD, standard deviation; MMR, mismatch repair; NR, not reported; CC, colon cancer.

### Survival analysis

A summary of all the survival analyses conducted is presented in *Table S4*. All included studies satisfied the proportional hazards assumption, except for one^30^. A single study retrieved survival from a per-protocol analysis (survival after surgery not from diagnosis)^24^. The survival data could not be extracted from the FOxTROT trial publication^29^; however, recognizing the importance of including these data for the analysis to be representative of the current evidence, these data were requested from the authors of the FOxTROT trial and were generously provided. After reconstructing, the patient-level survival data precisely aligned with the original published Kaplan–Meier values contained within the original articles (*Supplementary Results*).

#### Overall survival

**Fig. 2:**
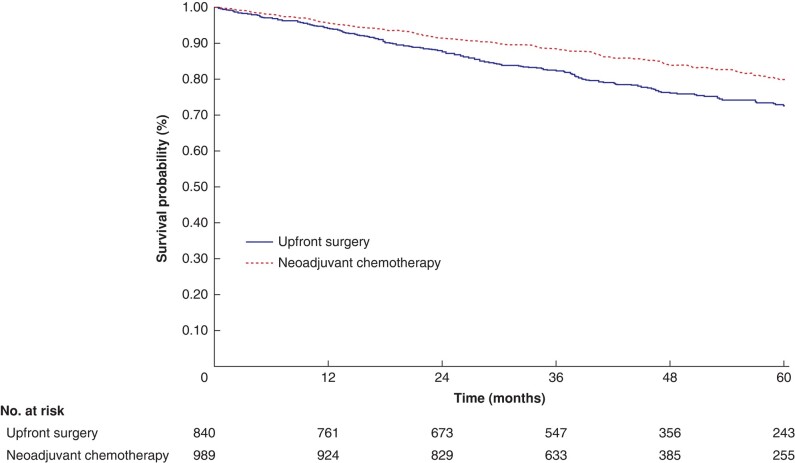
Kaplan–Meier overall survival plots depicting patients with locally advanced colon cancer categorized by treatment with neoadjuvant chemotherapy or upfront surgery

A total of five studies^23,24,26,28,29^ reported OS data encompassing a total of 1829 patients (989 in the NAC arm and 840 in the upfront surgery arm). The Kaplan–Meier-estimated OS rates at 1, 3, and 5 years were as follows: 97.7% (95% c.i. 89.5% to 96.8%), 88.5% (95% c.i. 86.3% to 90.4%), and 79.9% (95% c.i. 76.4% to 82.9%) for the NAC arm and 94.1% (95% c.i. 92.3% to 95.6%), 82.4% (95% c.i. 79.5% to 84.9%), and 72.6% (95% c.i. 68.8% to 76.0%) for the upfront surgery arm (*P* < 0.001) (*Fig. 2*). Based on the results of the marginal Cox regression analysis, NAC was associated with a statistically significant 33.5% decrease in the hazard of death compared with upfront surgery (HR 0.67, 95% c.i. 0.54 to 0.83; *P* < 0.001). In the two-stage meta-analysis, the pooled HR was 0.73 (95% c.i. 0.55 to 0.96; *P* = 0.022; *Figs S1, S2*).

#### Disease-free survival

**Fig. 3:**
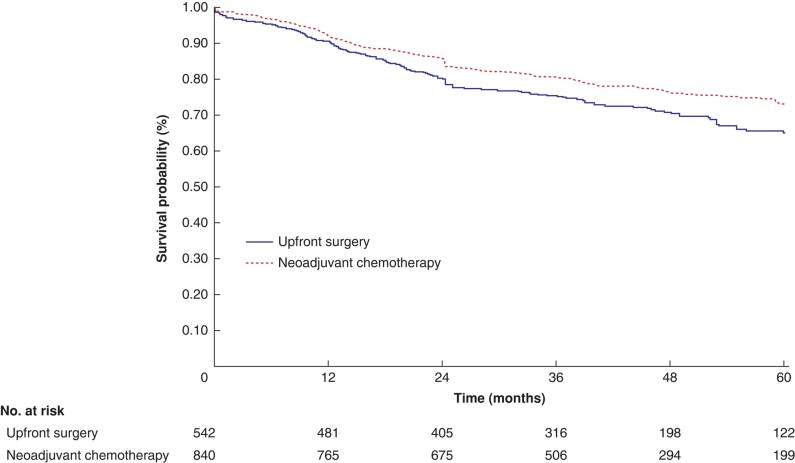
Kaplan–Meier disease-free survival plots depicting patients with locally advanced colon cancer categorized by treatment with neoadjuvant chemotherapy or upfront surgery

The DFS of 1382 patients with LACC was examined across four distinct studies^23,24,26,29^. Of these patients, 840 were assigned to NAC and 542 underwent upfront surgery. In the NAC group, the 1-, 3-, and 5-year DFS rates were 91.9% (95% c.i. 89.8% to 93.5%), 80.7% (95% c.i. 77.7% to 83.2%), and 73.1% (95% c.i. 69.2% to 76.5%) respectively. In the upfront surgery group, the corresponding rates at 1, 3, and 5 years were 90.6% (95% c.i. 87.8% to 92.8%), 75.4% (95% c.i. 71.5% to 78.9%), and 64.5% (95% c.i. 59.3% to 69.2%) respectively (*P* = 0.028) (*Fig. 3*).

Analysis using a Cox proportional hazards model demonstrated a significant 27.2% reduction in the hazard rate of recurrence associated with NAC (HR 0.73, 95% c.i. 0.59 to 0.90; *P* = 0.003). Additional survival analyses provided further support for the advantages of NAC (*Table S4*).

### Postoperative outcomes of surgical resection in locally advanced colon cancer

**Fig. 4:**
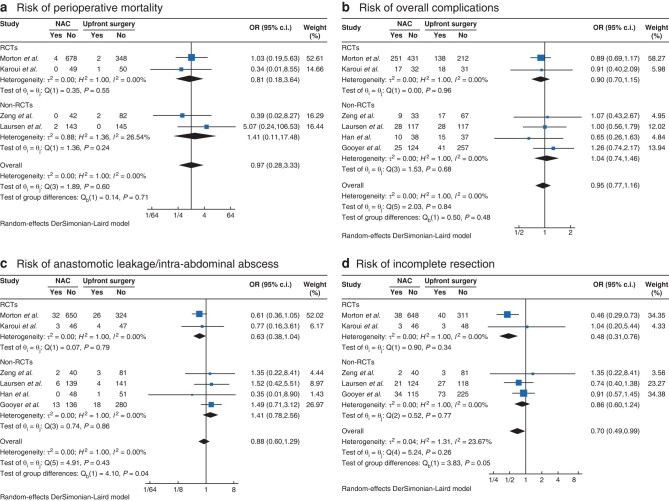
Forest plot depicting postoperative outcomes utilizing the DerSimonian and Laird model **a** Perioperative mortality. **b** Overall complications. **c** Anastomotic leakage/intra-abdominal abscess. **d** Incomplete resection.

A total of six studies involving 2095 patients with LACC were included in the analysis. Of the studies, four^24,25,27,29^ examined perioperative mortality, with no significant difference observed between the two treatment approaches (OR 0.97, 95% c.i. 0.28 to 3.33; *P* = 0.960; *Fig. 4a*).

Morbidity was assessed in all six studies^23–25,27–29^. There was no significant difference in the risk of overall complications between treatment arms (OR 0.95, 95% c.i. 0.77 to 1.16; *P* = 0.595; *Fig. 4b*). Similarly, the occurrence of anastomotic leakage/intra-abdominal abscess was not significantly different between the two groups (OR 0.88, 95% c.i. 0.60 to 1.29; *P* = 0.517; *Fig. 4c*). Patients in the NAC arm had a statistically significant 30% lower risk of incomplete resection (R1 or R2) compared with patients in the upfront surgery arm (OR 0.70, 95% c.i. 0.49 to 0.99; *P* = 0.045; *Fig. 4d*).

## Discussion

This meta-analysis focused on patients with radiologically staged T3–4 Nx–2 M0 potentially resectable LACC, demonstrating that NAC led to improved OS and DFS compared with upfront surgery followed by adjuvant chemotherapy. Additionally, NAC was associated with a lower risk of incomplete surgical resection. These survival associations are based on the analysis of original and reconstructed patient-level time-to-event data from high-quality studies and will be particularly relevant until the publication of conclusive results from ongoing RCTs, namely the FOxTROT 2 and 3 trials, as well as the ELECLA, OPTICAL, and Danish trials^31–34^.

For patients diagnosed with LACC, the current standard of care involves surgery followed by adjuvant chemotherapy with CAPOX for 3 months or an alternative 3–6 month course of FOLFOX^35^. In the context of stage III CC, patients treated with this approach are reported to have 5-year OS rates ranging from 60% to 80%^36,37^. Additionally, the 5-year DFS rate for stage III CC is generally reported to be around 60%^38,39^. However, NAC is increasingly being adopted in the management of LACC due to additional advantages over the standard approach. First, NAC provides valuable information regarding the efficacy of the chemotherapy agents employed, enabling an assessment of tumour response and a greater understanding of disease biology. Second, NAC enables the administration of systemic treatment to a larger cohort of patients and is not influenced by postoperative complications. This is particularly important, as studies conducted for other types of cancer, such as pancreatic cancer, have demonstrated that postoperative complications are strongly associated with both a reduced disease-free interval and reduced OS. Importantly, such associations appear to be largely mediated by the omission of adjuvant chemotherapy^40^. Furthermore, a favourable response of the primary tumour to NAC translates into a reduction in tumour volume, which may facilitate minimally invasive surgery and is likely indicative of a similar effect on micrometastases, if present. The application of a minimally invasive approach, even for T4 tumours, has demonstrated enhanced outcomes, by lowering mortality and complications, while maintaining oncological effectiveness^41^. Toxicity is not consistently reported across the studies; however, NAC appears to have acceptable tolerance, as indicated by commendably high completion rates.

Considering these benefits, NAC represents a promising alternative to standard treatment. However, current clinical guidelines only propose the use of NAC as an alternative treatment option for cT4 or CC with a high nodal burden^2,4,7^. This can be attributed to the previously limited quality of published evidence and, in particular, the scarcity of data on survival. The decision to recommend NAC over the current treatment standard will require the demonstration of a survival advantage after treatment with NAC, hence the rationale for the present meta-analysis.

The findings of the present study indicate that NAC is not associated with a significant increase in morbidity, mortality, or specific complications, such as anastomotic leakage. These findings indicate that surgery after NAC is a safe and viable treatment alternative. Furthermore, the present study reveals an association between the use of NAC and improvements in long-term survival, which is plausible and consistent with the benefits of NAC from a biological perspective. The observed benefit of NAC with regards to DFS is likely attributable to the tumour response and down-staging, leading to a higher rate of complete resection and tumour-free margins (R0). It is well documented that the presence of involved resection margins (R1/R2) in patients with LACC significantly reduces DFS compared with complete resection^42^. The present analysis revealed that NAC is associated with a 30% reduced risk of incomplete resection compared with upfront surgery, which undoubtedly contributes to improved DFS. Furthermore, the present study demonstrates an association between NAC and improvements in OS. The OS curves (*Fig. 2*) initially exhibit parallel and equal trajectories for the first 12 months, after which they progressively diverge. This notable difference may be attributed to delayed recurrence after treatment with NAC, particularly in patients with high-risk tumours.

The scientific community has eagerly awaited definitive evidence on the efficacy of NAC in the treatment of LACC for over a decade^43^. The findings of the present study represent a significant advancement towards a paradigm shift in the management of LACC. These results are in line with several retrospective studies and provide more precise and accurate estimates of long-term survival and treatment effects^44–47^. The observed survival benefit associated with NAC serves as a crucial factor supporting its use over the current standard of care. Furthermore, the inclusion of a substantial proportion of patients with T3 disease in the present study highlights the benefits of NAC specifically in this subgroup, justifying a potential extension of the existing treatment guidelines^2,4,7^. The robustness of these findings will be further established with the results of ongoing RCTs, which will provide fundamental insights into survival outcomes. Until these results become available, the present study provides an important contribution to the understanding of the efficacy of NAC in the treatment of LACC.

Over the course of the present study, two significant RCTs comparing NAC with upfront surgery in patients with LACC were presented at ASCO 2022 and 2023 Meeting, and were subsequently published in abstract form^33,34^. To maintain consistency with the inclusion/exclusion criteria of the present study, these trials were not included in the primary analysis. However, acknowledging the crucial significance of these phase III trials, a supplementary analysis incorporating these two trials alongside the studies included in the primary analysis was conducted, yielding results (for almost 3000 patients) that further emphasize the advantages of NAC for both OS and DFS. In recognition of the substantial contribution made by these trials (OPTICAL and NeoCol), the authors have chosen to incorporate the entire analysis in *Appendix S4*.

The present study represents a comprehensive collection and analysis of the most reliable and high-quality published studies available on NAC in LACC. To the authors’ knowledge, the present study is the first meta-analysis in this field to utilize reconstructed patient-level and original individual participant survival data, representing the highest level of published evidence to date. However, it is important to acknowledge a limitation of the present study, namely the potential for selection bias. This limitation arises due to discrepancies between the included propensity score-matched studies in terms of the variables used for matching, calliper widths, and approaches to handling missing data. The utilization of diverse propensity score-matching methods may also bias effect estimates. To address this concern, efforts were made to ensure comparability among the studies in terms of sample size, study design, and relevant characteristics. Certainly, the inclusion of RCTs along with other types of studies could potentially have introduced heterogeneity, resulting in variations across methodologies, patient populations, and interventions. To address this issue, sensitivity analyses were conducted, separating RCTs and non-RCTs (Figs S2–S6). A further sensitivity analysis was performed to exclude one study employing a per-protocol survival analysis; however, the results were consistent with the primary analysis (Figs S7–S9).

It is also important to acknowledge that this meta-analysis encompasses a variety of NAC regimens, including the incorporation of monoclonal antibodies. Future clinical trials should consider incorporating surgical parameters, such as the extent of lymphadenectomy or the attainment of complete mesocolic excision, as these factors, while not consistently reported, could have potential implications for survival outcomes. It is also important to acknowledge that the studies incorporated in the present analysis did not specifically capture information on tumour location, mismatch repair, or mutational status as selection criteria. These factors could potentially influence a tumour’s response to NAC and have an impact on survival. A further consideration is the need for accurate radiological staging to accurately identify patients who are suitable candidates for NAC^48,49^. The exploration of novel radiological findings, such as the emerging small arteriole sign, warrants meticulous consideration^50^.

The results of the present study demonstrate that treatment with NAC is associated with a reduced hazard of recurrence and death, as well as improved long-term OS and DFS rates, compared with upfront surgery in patients with LACC. Notably, administration of NAC did not lead to an increase in postoperative morbidity or mortality, but significantly increased the proportion of patients with complete resection. These results demonstrate that NAC presents a secure, efficient, and satisfactory oncological treatment strategy for patients with LACC.

## Supplementary Material

znae021_Supplementary_Data

## Data Availability

Patient-level deidentified survival data were extracted from articles published in peer-reviewed journals. Original survival data from the FOxTROT trial were requested from the authors. Survival data extracted and used in this study can be requested by contacting the corresponding author. FOxTROT trial data are not available for sharing and should be requested from the FOxTROT trial authors.
