# Supplementary material for: Survival and safety after neoadjuvant chemotherapy or upfront surgery for locally advanced colon cancer: meta-analysis
Source: Br J Surg. 2024 Feb 21;111(2):znae021. doi: 10.1093/bjs/znae021 (PMC10881053; doi:10.1093/bjs/znae021)
Supplement: znae021_Supplementary_Data [file znae021_supplementary_data.zip › Appendix_NAT_LAST (12-23)docx.docx]

**SURVIVAL AND SAFETY AFTER NEOADJUVANT CHEMOTHERAPY OR UPFRONT SURGERY FOR LOCALLY ADVANCED COLON CANCER: A PATIENT-LEVEL META-ANALYSIS**

Daniel Aliseda MD^1^, Jorge Arredondo PhD ^1,2^, Carlos Sánchez-Justicia MD^1,2^, Alicia Alvarellos MD^1^, Javier Rodríguez PhD^2,3^, Ignacio Matos PhD^2,3^, Fernando Rotellar PhD^1,2^, Jorge Baixauli PhD^1,2^, Carlos Pastor PhD^1,2^.

1. Department of General Surgery, Division of Colorectal Surgery. Clinica Universidad de Navarra, University of Navarra, Pamplona-Madrid, Spain.

2. Institute of Health Research of Navarra (IdisNA), Pamplona, Spain.

3. Department of Oncology, Clinica Universidad de Navarra, University of Navarra, Pamplona-Madrid, Spain.

**Corresponding author:** Jorge Arredondo. Department of General Surgery, Clinica Universidad de Navarra, University of Navarra, Av. Pío XII, 36, 31008, Pamplona, Spain. Phone number: 34 948255400-ext 4711-. e-mail: [jarredon@unav.es](mailto:jarredon@unav.es).

**SUPPLEMENTARY INDEX**

[SUPPLEMENTARY RESULTS 4](#_Toc152603377)

[Patient-Level Survival Reconstruction 4](#_Toc152603378)

[Survival analysis 20](#_Toc152603379)

[Publication bias 26](#_Toc152603380)

[SUPPLEMENTARY TABLES 29](#_Toc152603381)

[Table 1S. Studies excluded from the analysis 29](#_Toc152603382)

[Table 2S. Risk of bias assessment for the included studies 30](#_Toc152603383)

[Table 3S. Newcastle–Ottawa score for the included studies 31](#_Toc152603384)

[Table 4S. Survival analysis using reconstructed patient-level data survival information. 32](#_Toc152603385)

[SUPPLEMENTARY FIGURES 33](#_Toc152603386)

[Figure 1S. Forest plot illustrating the two-stage meta-analysis for overall survival using a random effects meta-analysis. 33](#_Toc152603387)

[Figure 2S. Forest plot illustrating the two-stage meta-analysis for overall survival using a random effects meta-analysis (separating RCTs and Non-RCTs studies). 33](#_Toc152603388)

[Figure 3S. Forest plot illustrating the two-stage meta-analysis for disease-free survival using a random effects meta-analysis. 34](#_Toc152603389)

[Figure 4S. Forest plot illustrating the two-stage meta-analysis for disease-free survival using a random effects meta-analysis (separating RCTs and Non-RCTs studies). 34](#_Toc152603390)

[Figure 5S. Kaplan-Meier OS plots depicting patients with LACC categorized by treatment with NAC or upfront surgery and separated by study design (RCT and no RCTs). 35](#_Toc152603391)

[Figure 6S. Kaplan-Meier DFS plots depicting patients with LACC categorized by treatment with NAC or upfront surgery and separated by study design (RCT and no RCTs). 36](#_Toc152603392)

[Figure 7S. Forest plot illustrating the two-stage meta-analysis for disease-free survival using a random effects meta-analysis (excluding studies with per protocol analysis). 37](#_Toc152603393)

[Figure 8S. Kaplan-Meier OS plots depicting patients with LACC categorized by treatment with NAC or upfront surgery and separated by study design (excluding studies with per protocol analysis). 37](#_Toc152603394)

[Figure 9S. Kaplan-Meier DFS plots depicting patients with LACC categorized by treatment with NAC or upfront surgery and separated by study design (excluding studies with per protocol analysis). 38](#_Toc152603395)

[SUPPLEMENTARY APPENDIXES 39](#_Toc152603396)

[Search strategy 39](#_Toc152603397)

[Prospero registered protocol 40](#_Toc152603398)

[Prisma checklist 50](#_Toc152603399)

[Additional survival analysis 56](#_Toc152603400)

[*Optical trial (Asco 2022) 56*](#_Toc152603401)

[*NeoCol trial (Asco 2023) 62*](#_Toc152603402)

[*Final analysis disease free-survival (including Optical trial) 67*](#_Toc152603403)

[*Final analysis disease free-survival (including Optical and NeoCol trial) 69*](#_Toc152603404)

[*Final analysis overall survival (including NeoCol trial) 71*](#_Toc152603405)

[*Final analysis overall survival (including NeoCol and Optical trial) 73*](#_Toc152603406)

[REFERENCES 75](#_Toc152603407)

# SUPPLEMENTARY RESULTS

## PATIENT-LEVEL SURVIVAL RECONSTRUCTION

Morton et al (2023)^1^

Grambsch–Therneau test

*Overall survival*

**
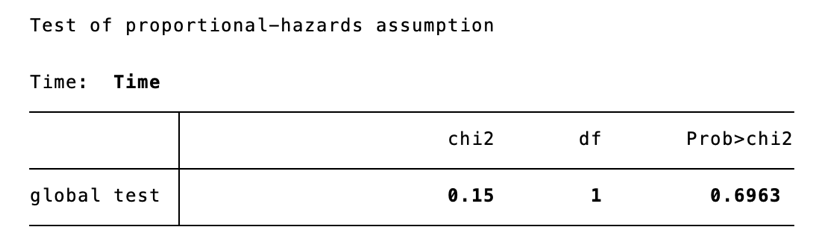
**

*Disease-free survival*

**
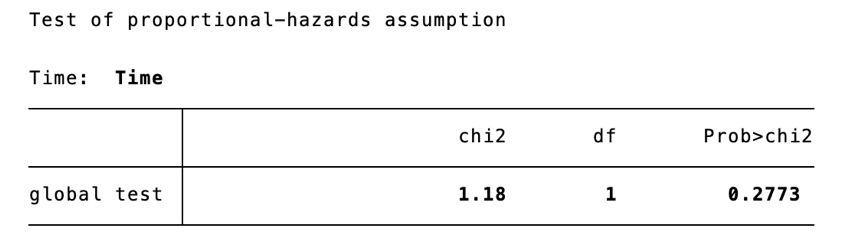
**

Schoenfeld residuals plot

*Overall survival*

*Disease-free survival*

Predicted versus observed survivor functions

*Overall survival*

*Disease-free survival*

Zeng et al. (2022)^2^

**OVERALL SURVIVAL**

**
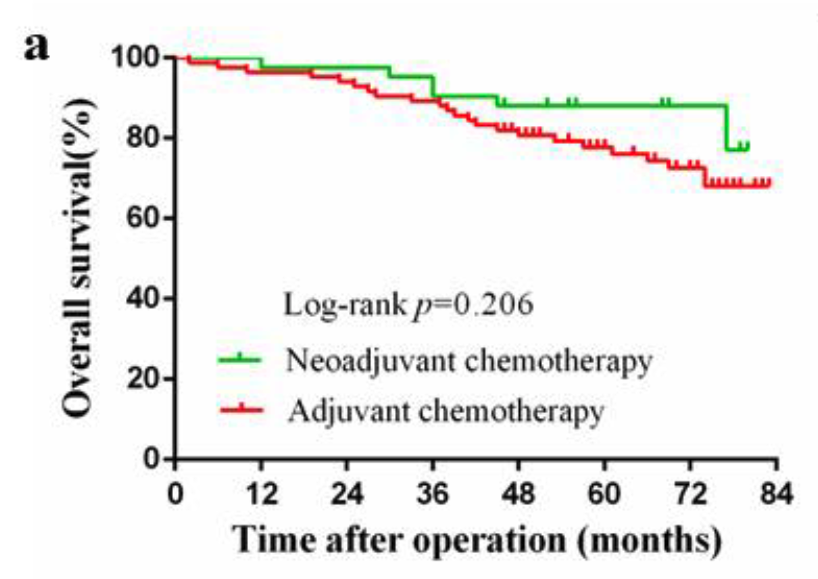
**Kaplan–Meier curve from original paper

Reconstructed survival curves including log-rank test

**
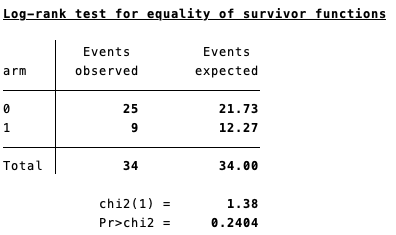
**

**DISEASE FREE-SURVIVAL**

Kaplan–Meier curve from original paper

**
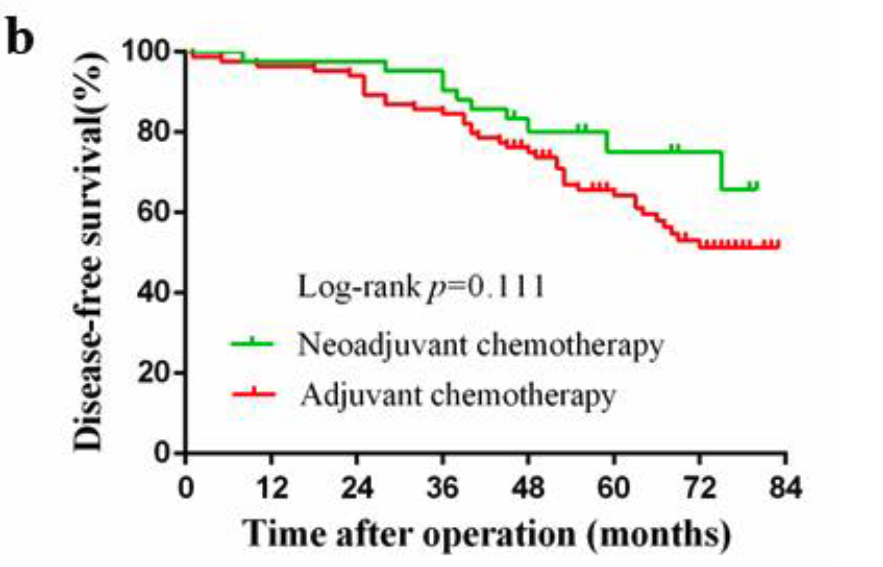
**

Reconstructed survival curves including log-rank test

**
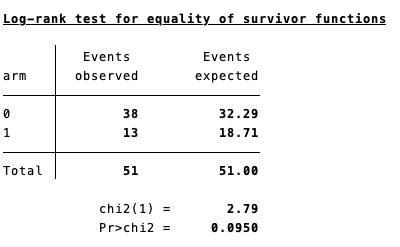
**

Grambsch–Therneau test

*Overall survival*

**
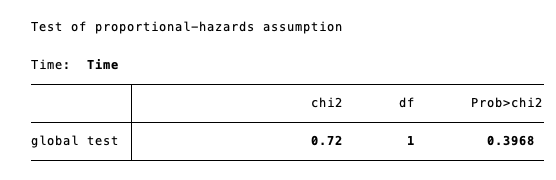
**

*Disease-free survival*

**
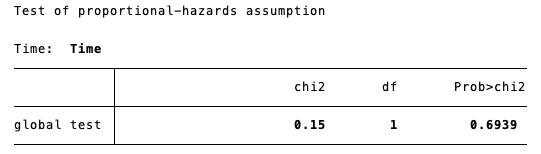
**

Schoenfeld residuals plot

*Overall survival*

*Disease-free survival*

Predicted versus observed survivor functions

*Overall survival*

*Disease-free survival*

Han et al. (2022)^3^

**OVERALL SURVIVAL**

Kaplan–Meier curve from original paper


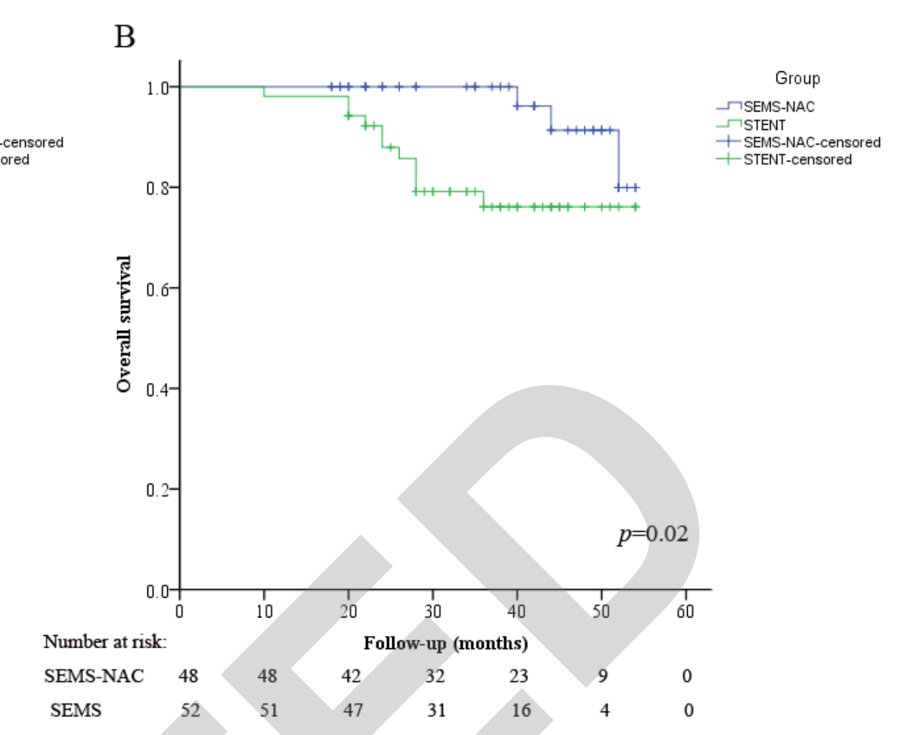


Reconstructed survival curves including number-at-risk tables


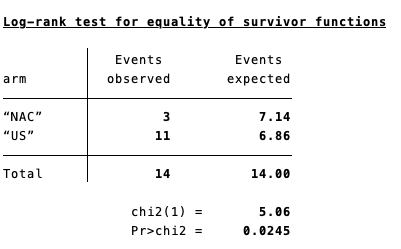


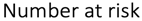


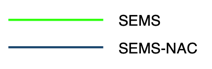


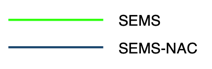


**DISEASE FREE-SURVIVAL**

Kaplan–Meier curve from original paper


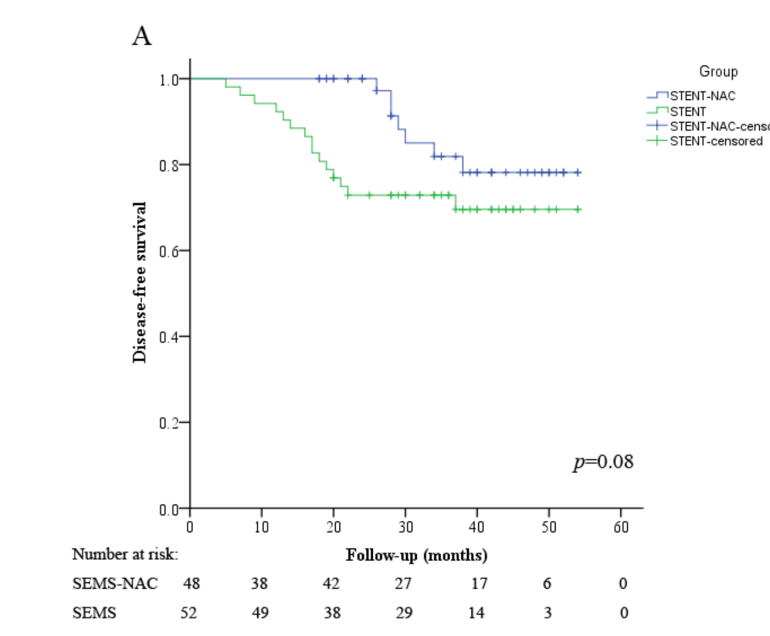


Reconstructed survival curves including number-at-risk tables


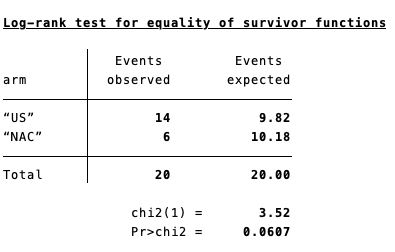


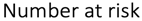

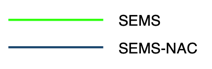


Grambsch–Therneau test

*Overall survival*


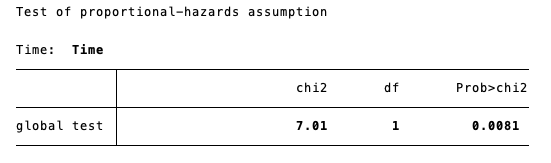


*Disease-free survival*


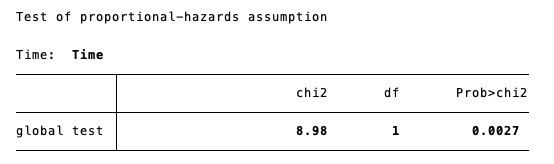


Schoenfeld residuals plot

*Overall survival*

*Disease-free survival*

Predicted versus observed survivor functions

*Overall survival*

*Disease-free survival*

Karoui et al. (2021)^4^

**OVERALL SURVIVAL**

Kaplan–Meier curve from original paper


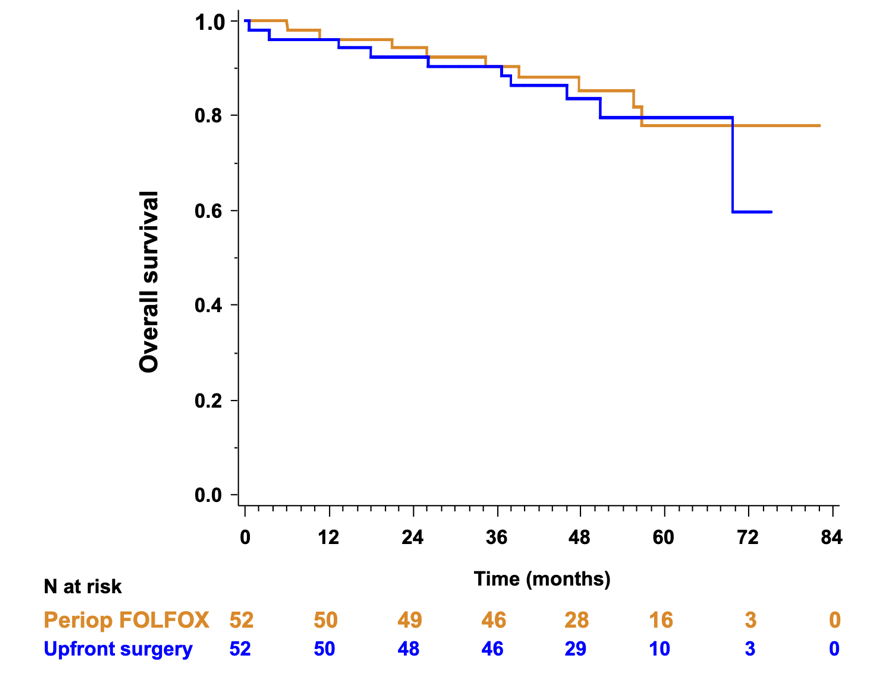


Reconstructed survival curves

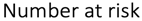


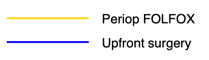


**DISEASE-FREESURVIVAL**

Kaplan­Meier curve from original paper

**
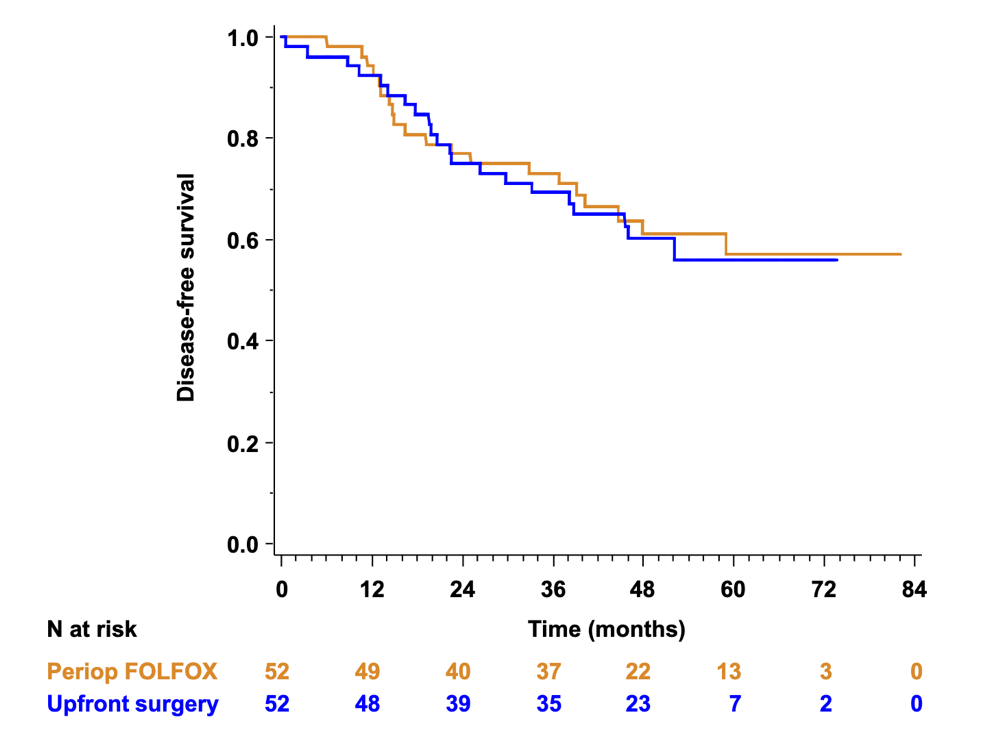
**

Reconstructed survival curves

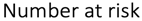


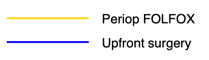


Grambsch–Therneau test

*Overall survival*


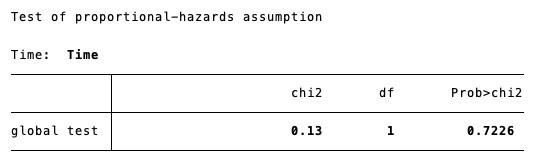


*Disease-free survival*


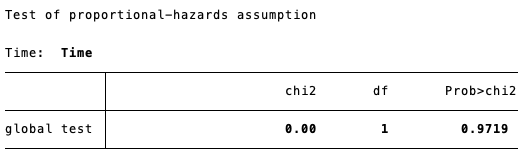


Schoenfeld residuals plot

*Overall survival*

*Disease-free survival*

Predicted versus observed survivor functions

*Overall survival*

*Disease-free survival*

De Gooyer et al (2020)^5^

**OVERALL SURVIVAL**

Kaplan–Meier curve from original paper


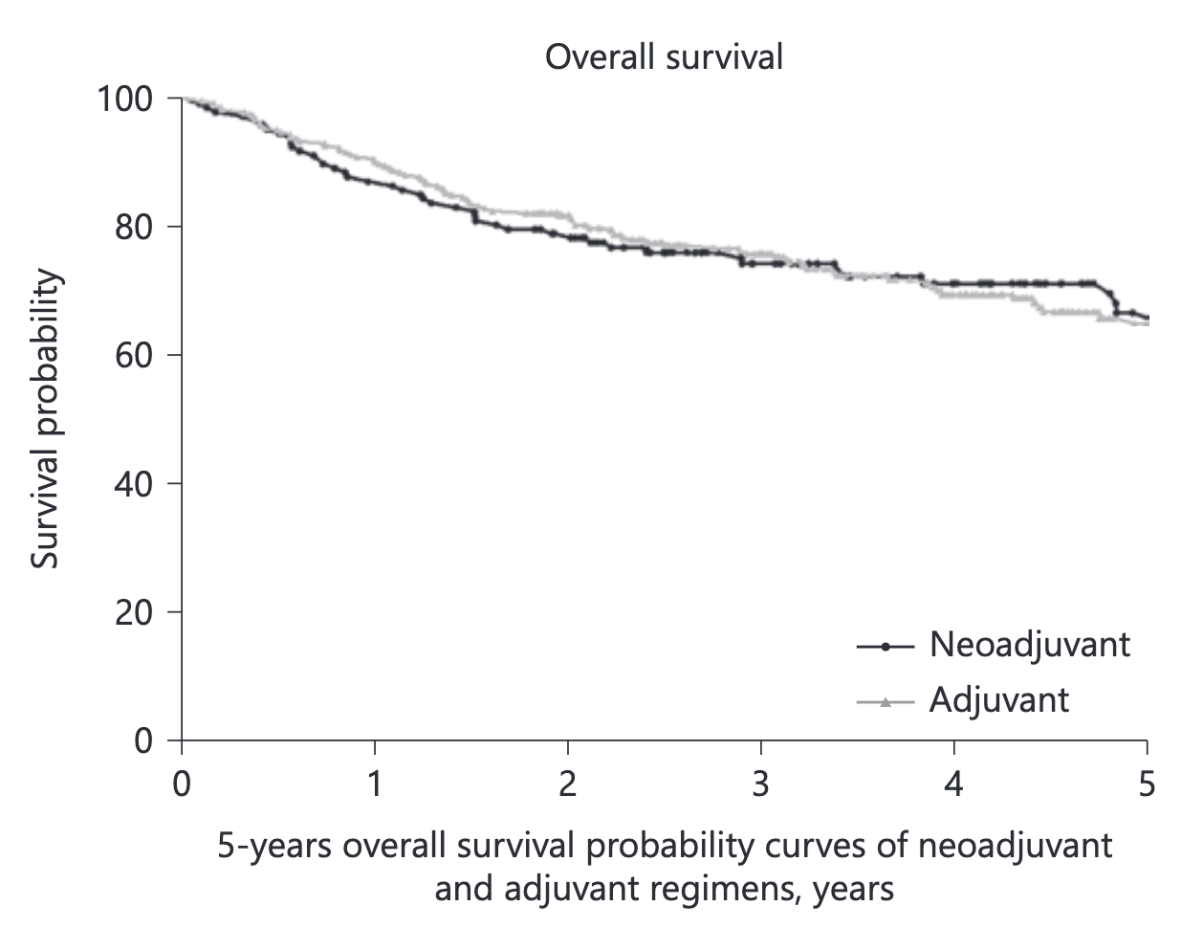


Reconstructed survival curves

Grambsch–Therneau test


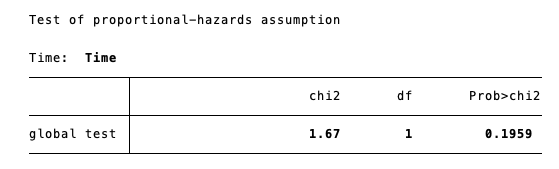


Schoenfeld residuals plot

Predicted versus observed survivor functions

Log-rank test

**
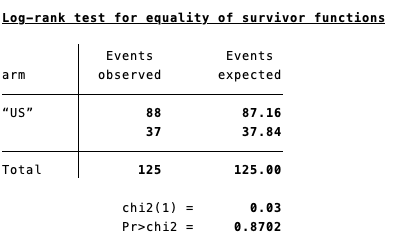
**

## SURVIVAL ANALYSIS

Overall survival

Reconstructed survival curves including number-at-risk tables


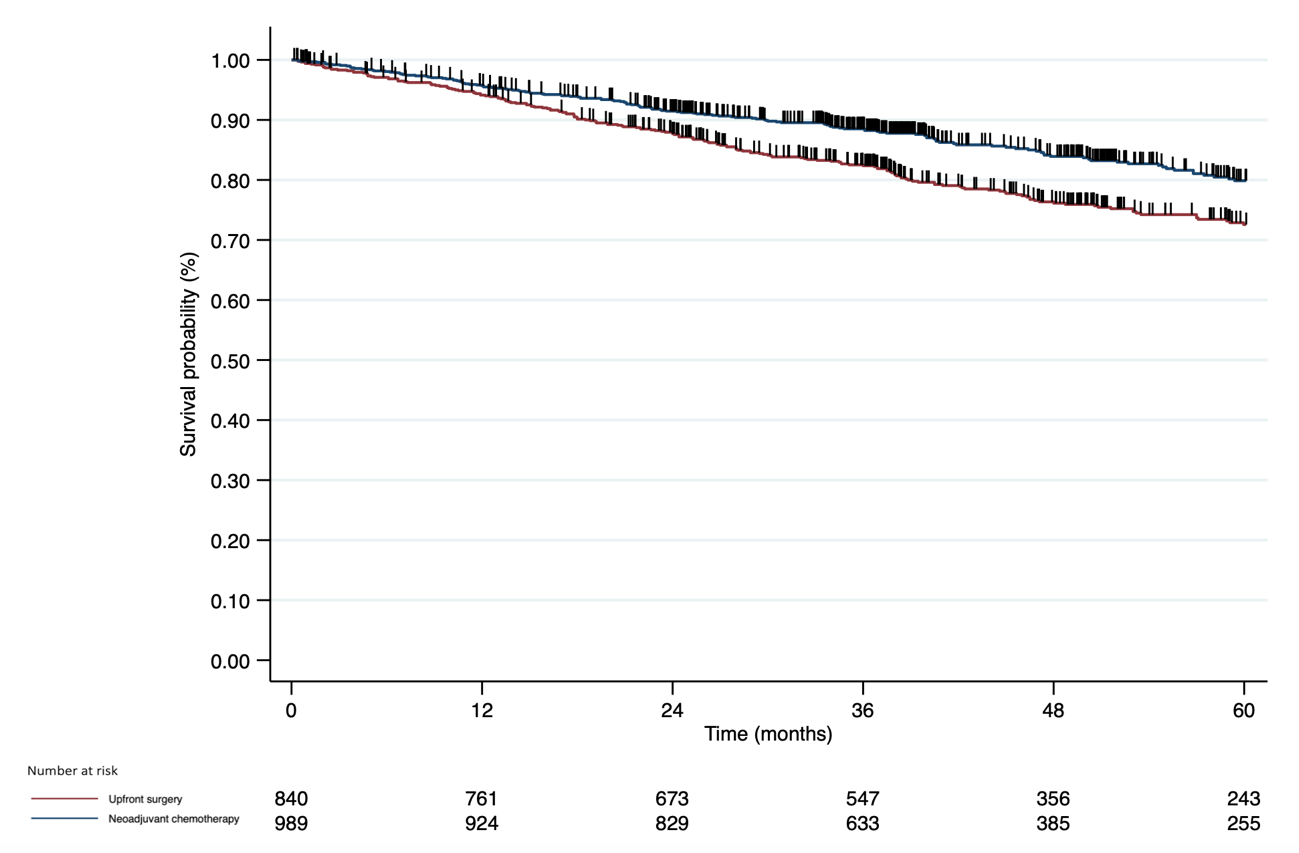


Grambsch–Therneau test


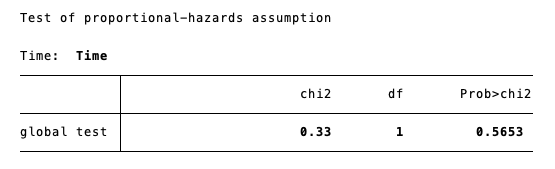


Schoenfeld residuals plot

Predicted versus observed survivor functions

Marginal Cox regression


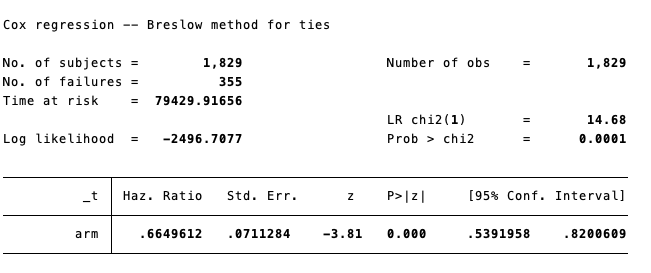


Disease-free survival

Reconstructed survival curves including number-at-risk tables


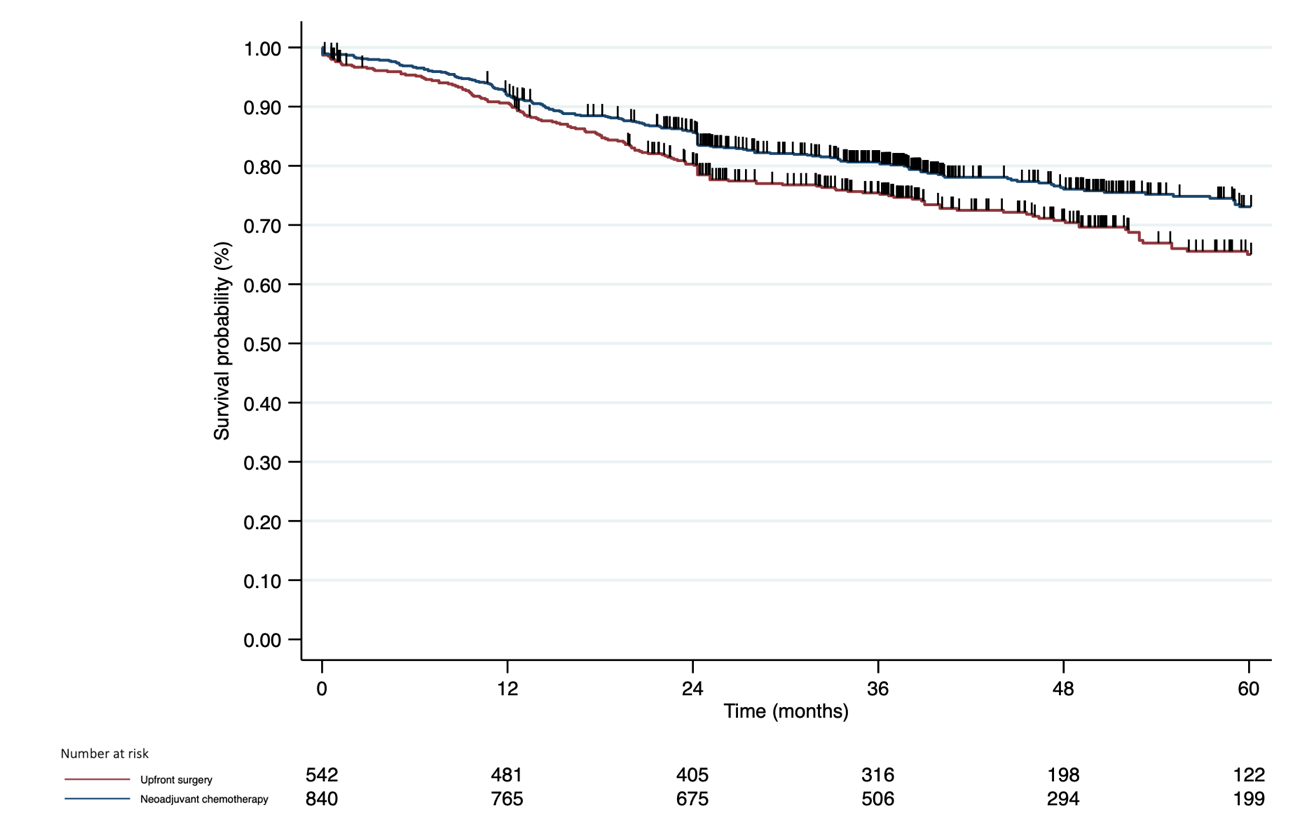


Grambsch–Therneau test


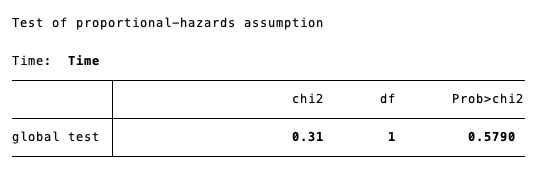


Schoenfeld residuals plot

Predicted versus observed survivor functions

Marginal Cox regression

**
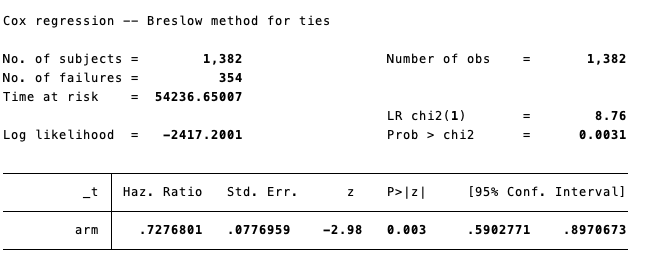
**

## PUBLICATION BIAS

Funnel plot showing publication bias; peri-operative mortality (X-axis, log OR) with standard error (Y-axis)

Funnel plot showing publication bias; overall complications (X-axis, log OR) with standard error (Y-axis)

Funnel plot showing publication bias; anastomotic leak or abscess (X-axis, log OR) with standard error (Y-axis)

Funnel plot showing publication bias; complete resection (R0) (X-axis, log OR) with standard error (Y-axis)

Funnel plot showing publication bias; hazard ratio (X-axis) with standard error (Y-axis)

**OS**

**DFS**

| **Table 1S.** Studies excluded from the study | | |
| --- | --- | --- |
| **Study** | **PMID** | **Cause of exclusion** |
| Safety and feasibility of neo-adjuvant chemotherapy as a surgical bridge for acute left‑sided malignant colorectal obstruction: a retrospective study | **35864459** | Includes rectal tumours |
| Neo-adjuvant radiation for clinical T4 colon cancer: A potential improvement to overall survival | **30017250** | Includes radiation therapy |
| Neo-adjuvant Radiation Therapy in Locally Advanced Colon Cancer: a Cohort Analysis | **29427227** | Includes radiation therapy |
| Clinical efficacy of capecitabine combined with oxaliplatin on preoperative neo-adjuvant chemotherapy for colorectal cancer. | **-** | Impossibility to access the article |
| Neo-adjuvant chemotherapy for resectable locally advanced colon carcinoma: a prospective case controlled trial. | **-** | Impossibility to access the article |
| Results of a Randomized Trial with or without 5-FU-based Preoperative Chemotherapy followed by Postoperative Chemotherapy in Resected Colon and Rectal Carcinoma | **12913083** | Includes patients with non-locally advanced colon cancer |
| Incorporating neo-adjuvant chemotherapy into locally advanced colon cancer treatment pathways: Real-life experience of implementing FOxTROT | **3651112** | No control arm |
| A Prospective, Single-arm, Multi-center Trial of Diverting Stoma Followed by Neoadjuvant Chemotherapy Using mFOLFOX6 for Obstructive Colon Cancer | **32941273** | No control arm |
| Does preoperative neo-adjuvant chemotherapy impact short-term surgical outcomes in patients with locally advanced colon cancer? | **33963914** | Retrospective non-matched study |
| Is neoadjuvant therapy an alternative strategy to immediate surgery in locally perforated colon cancer? | **34379861** | No control arm |
| Outcomes of neo-adjuvant chemoradiotherapy followed by radical resection for T4 colorectal cancer | **33362913** | Includes radiation therapy  Includes rectal tumours |
| Neo-adjuvant Chemotherapy Improves Survival in Patients with Clinical T4b Colon Cancer | **28933016** | Insufficient survival data |
| Locally Advanced Colon Cancer: Evaluation of Current Clinical Practice and Treatment Outcomes at the Population Level | **28933016** | Retrospective non-matched study |
| A pilot phase II study of neo-adjuvant triplet chemotherapy regimen in patients with locally advanced resectable colon cancer | **28174488** | No control arm |
| Neo-adjuvant chemotherapy in locally advanced colon cancer. A phase II trial | **25920359** | Insufficient survival data |
| Preliminary outcome of a treatment strategy based on perioperative chemotherapy and surgery in patients with locally advanced colon cancer | **23398577** | No control arm |
| Feasibility of preoperative chemotherapy for locally advanced, operable colon cancer: the pilot phase of a randomised controlled trial | **23017669** | Duplicated data |

# SUPPLEMENTARY TABLES

## Table 1S. Studies excluded from the analysis

## Table 2S. Risk of bias assessment for the included studies


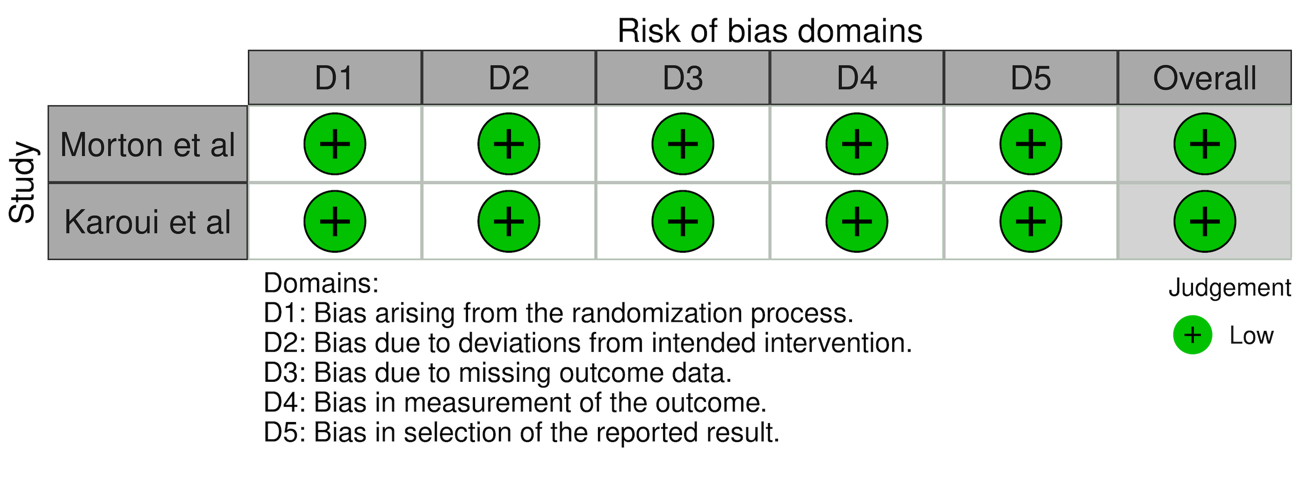


## Table 3S. Newcastle–Ottawa score for the included studies

| **Table 3S.** Newcastle–Ottawa score for the included studies | | | | | | | | | |
| --- | --- | --- | --- | --- | --- | --- | --- | --- | --- |
| **First author, year** | Representativeness of cohort | Selection of non-exposed cohort | Ascertainment of exposure | Demonstration that outcome of interest was not present at start of study | Comparability of cohorts on the basis of the design or analysis | Assessment of outcome | Was follow-up long enough for outcomes to occur | Adequacy of follow-up of cohorts | Total score |
| **Zeng et al. (2022)**^2^ | ★ | ★ | ★ | ★ |  | ★ | ★ | ★ | **7** |
| **Laursen et. al (2022)**^6^ | ★ | ★ | ★ | ★ | ★ | ★ | ★ | ★ | **8** |
| **Han et al. (2022)**^3^ | ★ | ★ | ★ | ★ |  | ★ | ★ | ★ | **7** |
| **Karoui et al. (2020)**^7^ | ★ | ★ | ★ | ★ | ★ | ★ | ★ | ★ | **8** |
| **Gooyer et al. (2020)** | ★ | ★ | ★ | ★ | ★ | ★ | ★ | ★ | **8** |

| **Table 4S.** Survival analysis using reconstructed patient-level data survival information | | | |
| --- | --- | --- | --- |
| **Survival Analysis** |  |  |  |
| **Overall survival** | **One stage meta-analysis** | **Effect size (95% CI)** | **P-value** |
|  | Marginal HR* | 0.665 (0.539–0.820) | **0.0001** |
|  | Stratified HR | 0.774 (0.619–0.968) | **0.0243** |
|  | Shared frailty HR | 0.766 (0.613–0.956) | **0.0186** |
|  | **“Two stage” meta-analysis** |  |  |
|  | HR (random-effects) | 0.728 (0.555–955) | **0.0218** |
|  | HR (random-effects) (excluding non-proportional hazards assumption) | 0.74 (0.61–0.90) |  |
|  | HR (random-effects) (separating RCTs and Non RCTs studies) | 0.68 (0.55–0.83) and 0.67 (0.36–1.26) |  |
|  | **Non-parametric models** |  |  |
|  | RMST difference (up to 5 year) | +2.9 months (1.382– 4.438) | **<0.0001** |
|  |  |  |  |
| **Disease-free survival** | **One stage meta-analysis** | **Effect size (95% CI)** | **P-value** |
|  | Marginal HR* | 0.728 (0.590–0.897) | **0.0031** |
|  | Stratified HR | 0.731 (0.590–0.906) | **0.0045** |
|  | Shared frailty HR | 0.728 (0.590–0.897) | **0.0029** |
|  | **“Two stage” meta-analysis** |  |  |
|  | HR (random-effects) | 0.715 (0.594–0.860) | **0.0004** |
|  | HR (random-effects) (excluding non-proportional hazards assumption) | 0.73 (0.60–0.88) |  |
|  | HR (random-effects) (separating RCTs and Non RCTs studies) | 0.75 (0.61–0.91) and 0.53 (0.31–0.90) |  |
|  | **Non-parametric models** |  |  |
|  | RMST difference (up to 5 year) | +2.8 months (0.742–4.866) | **0.008** |
| HR, hazard ratio; RMST, restricted median survival time; *Primary analysis; RCTs, Randomized controlled trials | | | |

## Table 4S. Survival analysis using reconstructed patient-level data survival information.

# SUPPLEMENTARY FIGURES

## Figure 1S. Forest plot illustrating the two-stage meta-analysis for overall survival using a random effects meta-analysis.

## Figure 2S. Forest plot illustrating the two-stage meta-analysis for overall survival using a random effects meta-analysis (separating RCTs and Non-RCTs studies).

## Figure 3S. Forest plot illustrating the two-stage meta-analysis for disease-free survival using a random effects meta-analysis.

## Figure 4S. Forest plot illustrating the two-stage meta-analysis for disease-free survival using a random effects meta-analysis (separating RCTs and Non-RCTs studies).

## Figure 5S. Kaplan-Meier OS plots depicting patients with LACC categorized by treatment with NAC or upfront surgery and separated by study design (RCT and no RCTs).


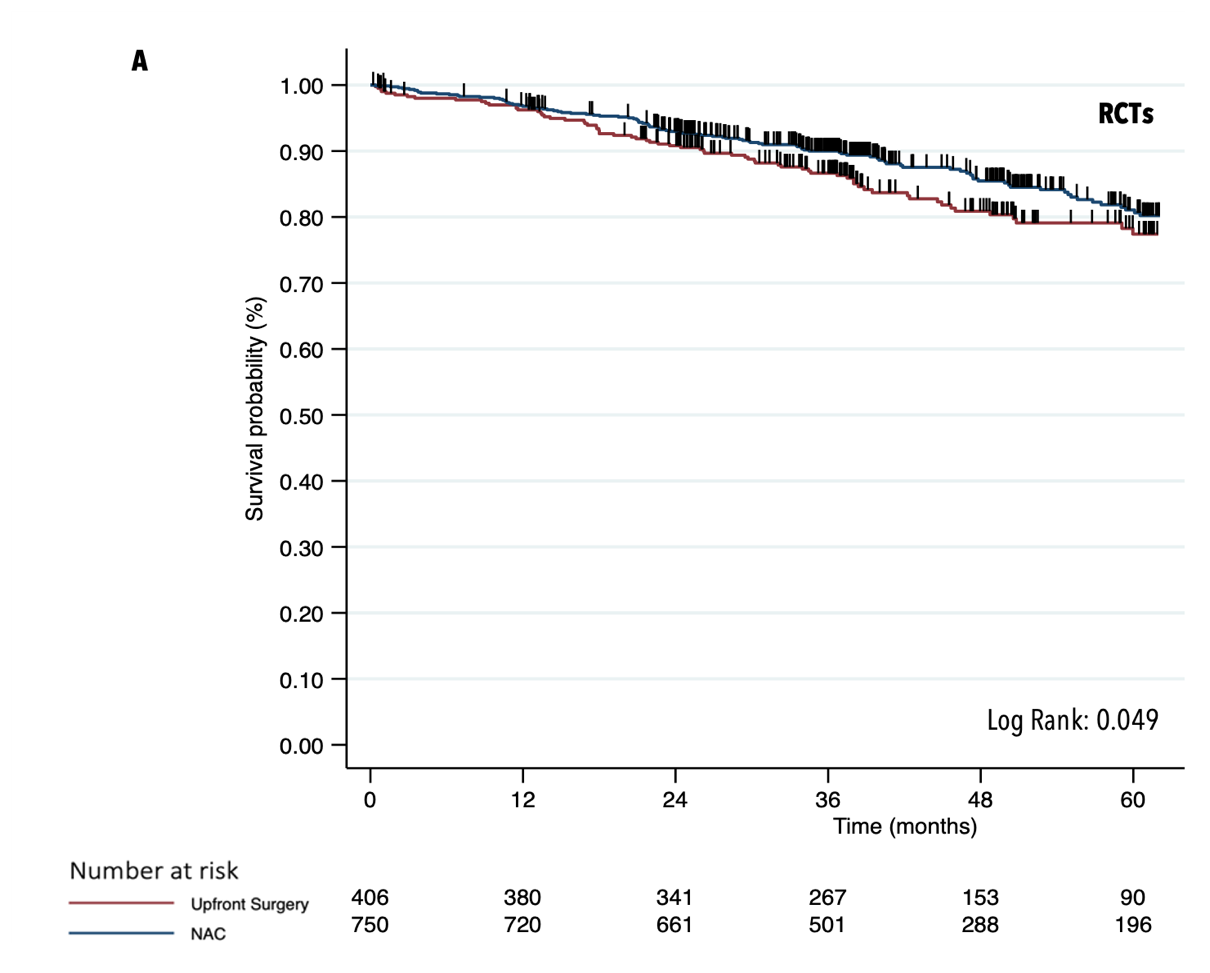


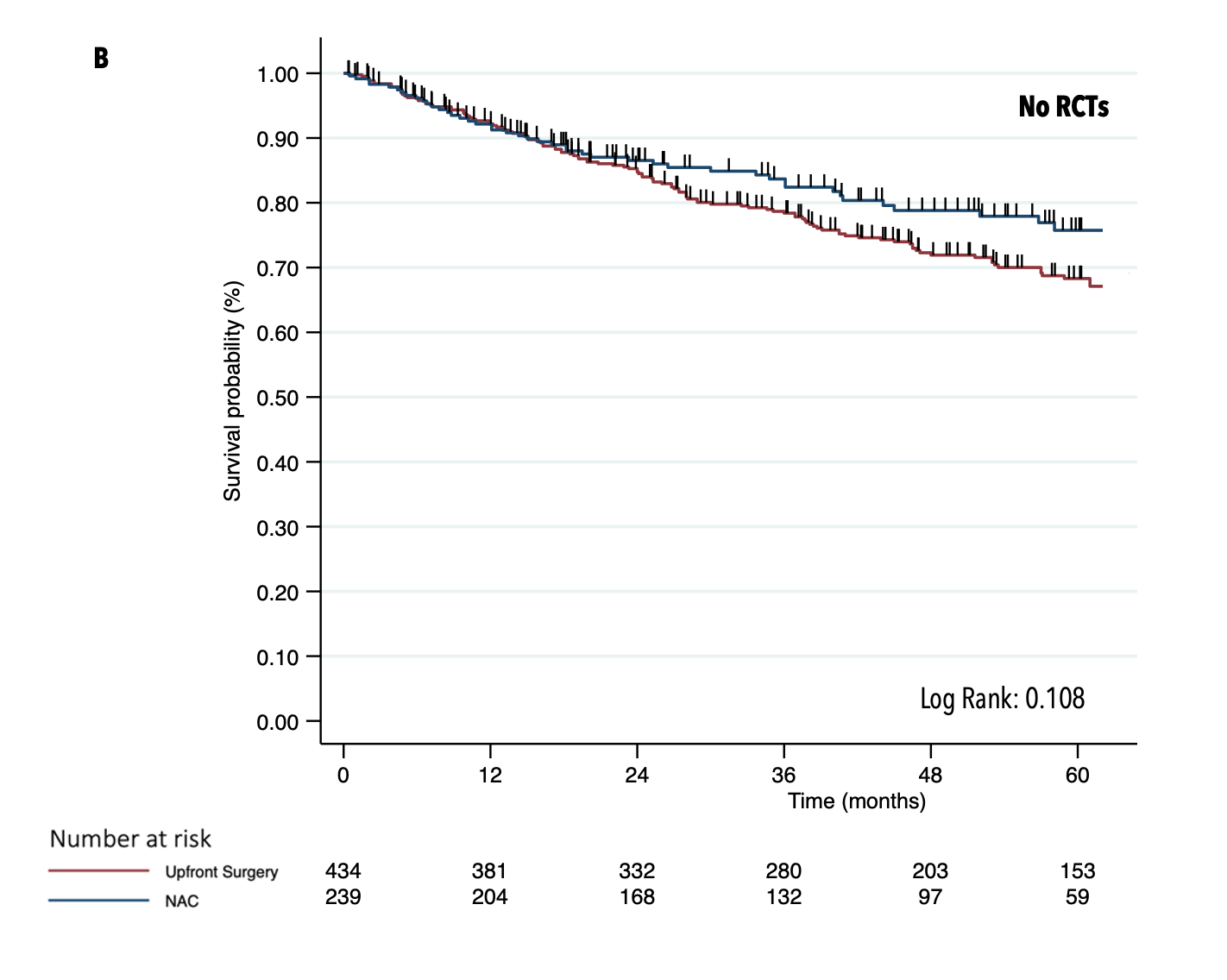


##
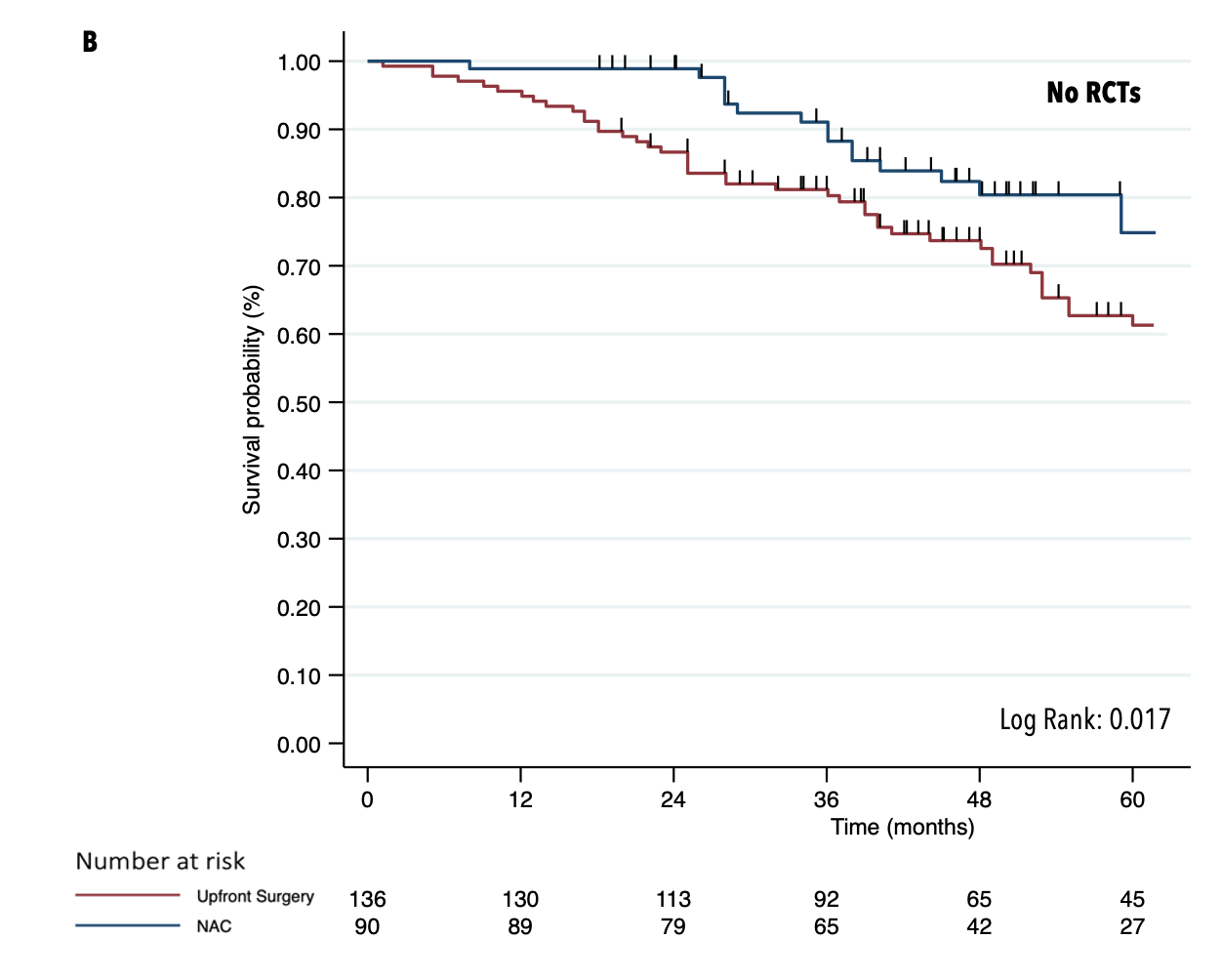

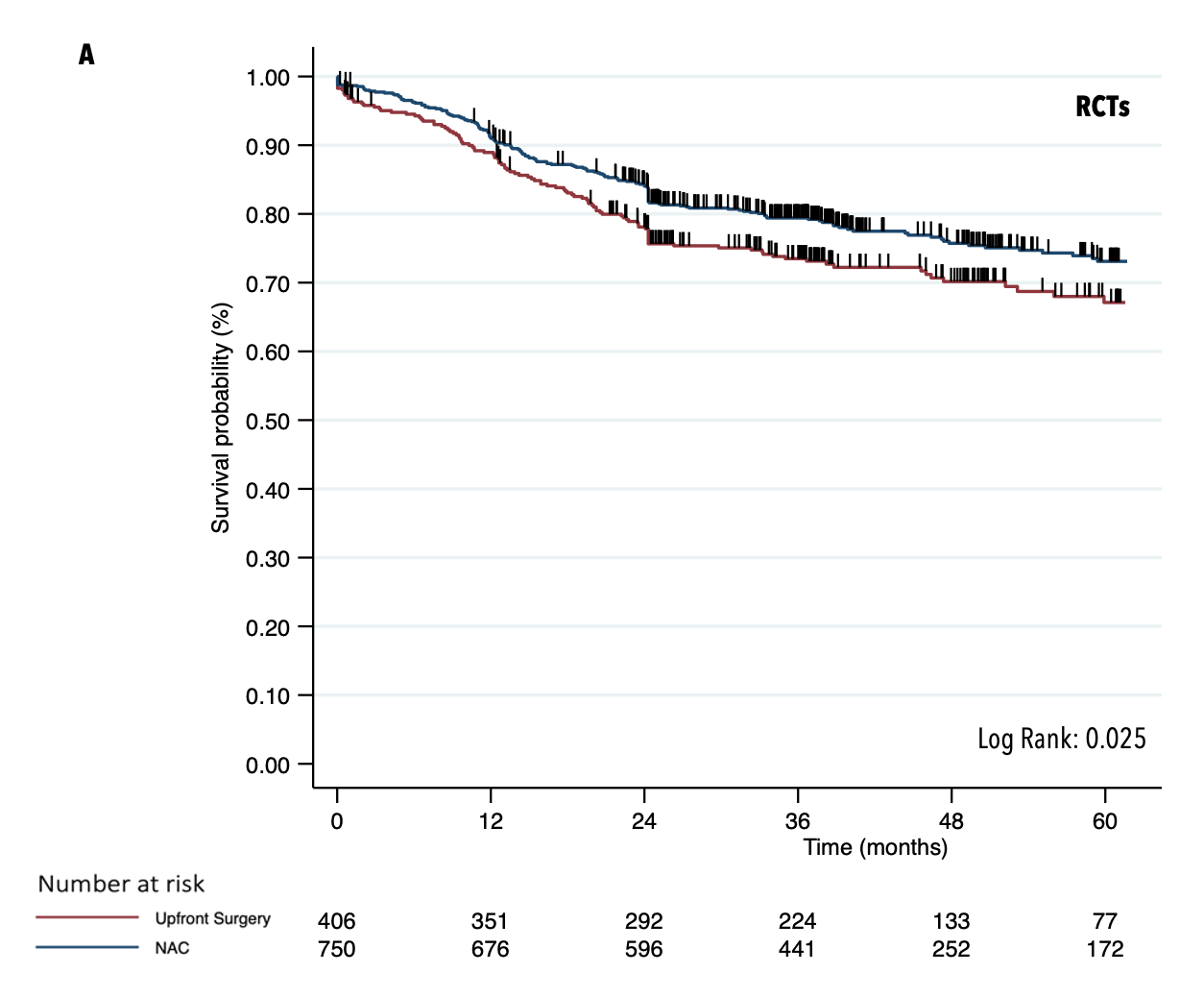
Figure 6S. Kaplan-Meier DFS plots depicting patients with LACC categorized by treatment with NAC or upfront surgery and separated by study design (RCT and no RCTs).

## Figure 7S. Forest plot illustrating the two-stage meta-analysis for disease-free survival using a random effects meta-analysis (excluding studies with per protocol analysis).

**A**

**B**

## Figure 8S. Kaplan-Meier OS plots depicting patients with LACC categorized by treatment with NAC or upfront surgery and separated by study design (excluding studies with per protocol analysis).


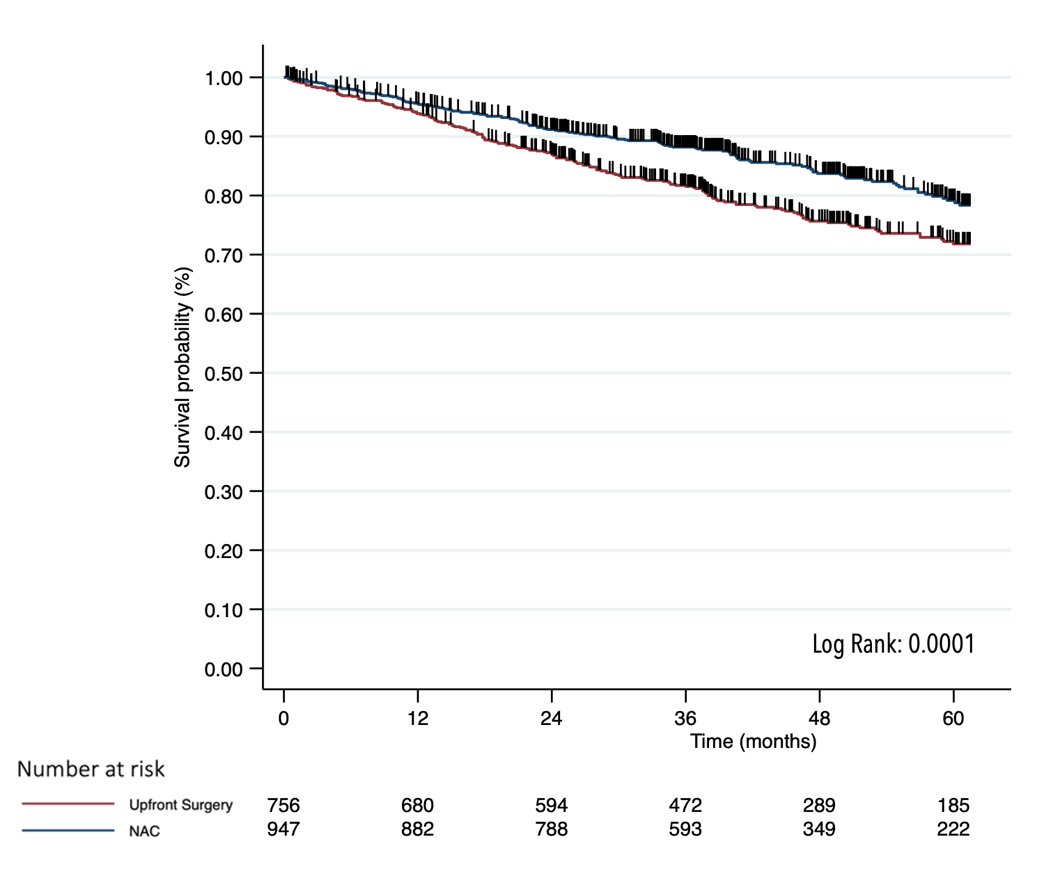


##

## Figure 9S. Kaplan-Meier DFS plots depicting patients with LACC categorized by treatment with NAC or upfront surgery and separated by study design (excluding studies with per protocol analysis).

##
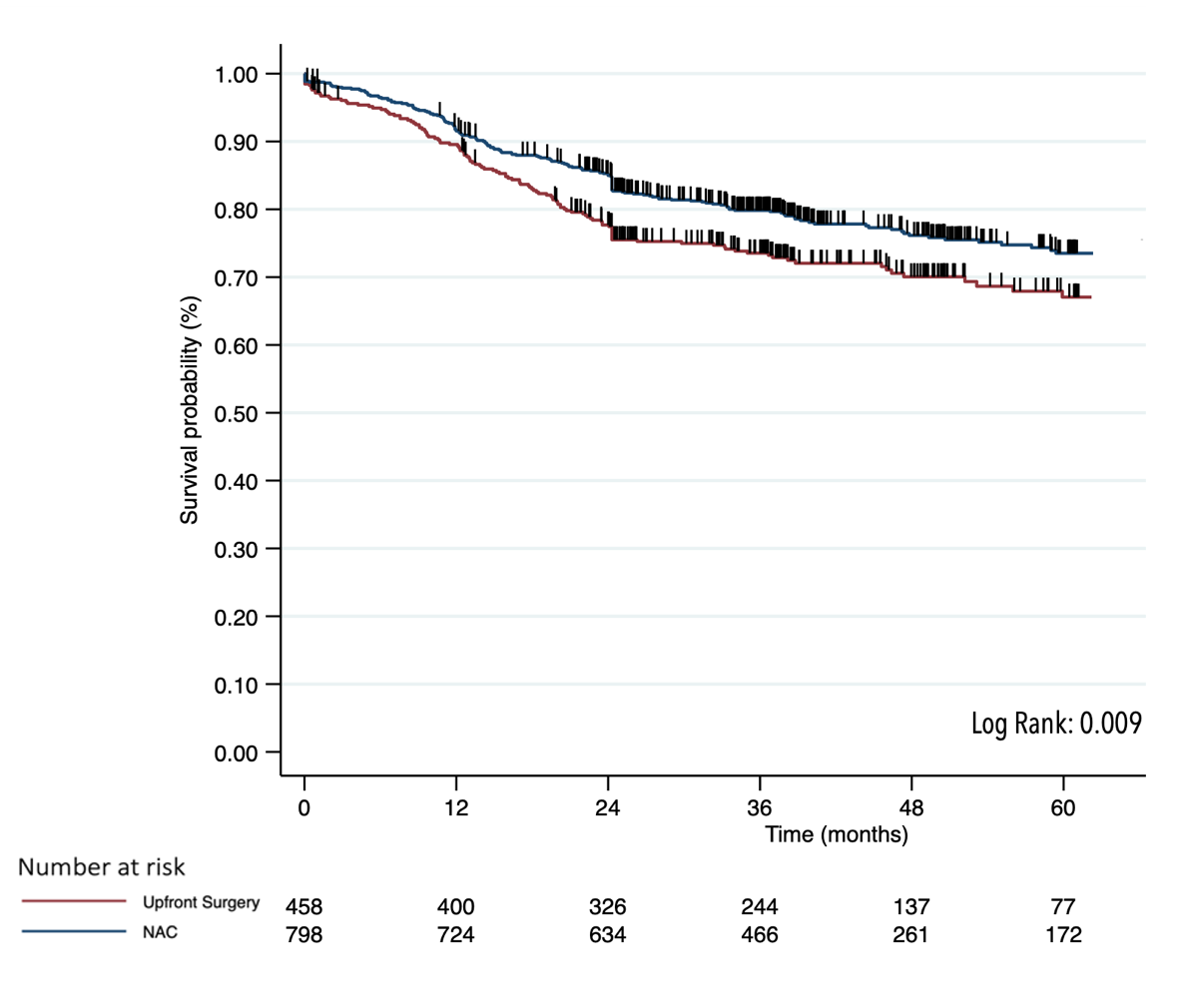


# SUPPLEMENTARY APPENDIXES

## SEARCH STRATEGY

Relevant studies were identified in Scopus and MEDLINE (via Ovid) using the following key terms: "colon cancer", "neoadjuvant", and "chemotherapy". All conceivable combinations of these keywords were utilized, and an additional cross-reference search of the included reports and other review articles was conducted. The study protocol was registered and subsequently updated on PROSPERO (CR42023422028).

## PROSPERO REGISTERED PROTOCOL


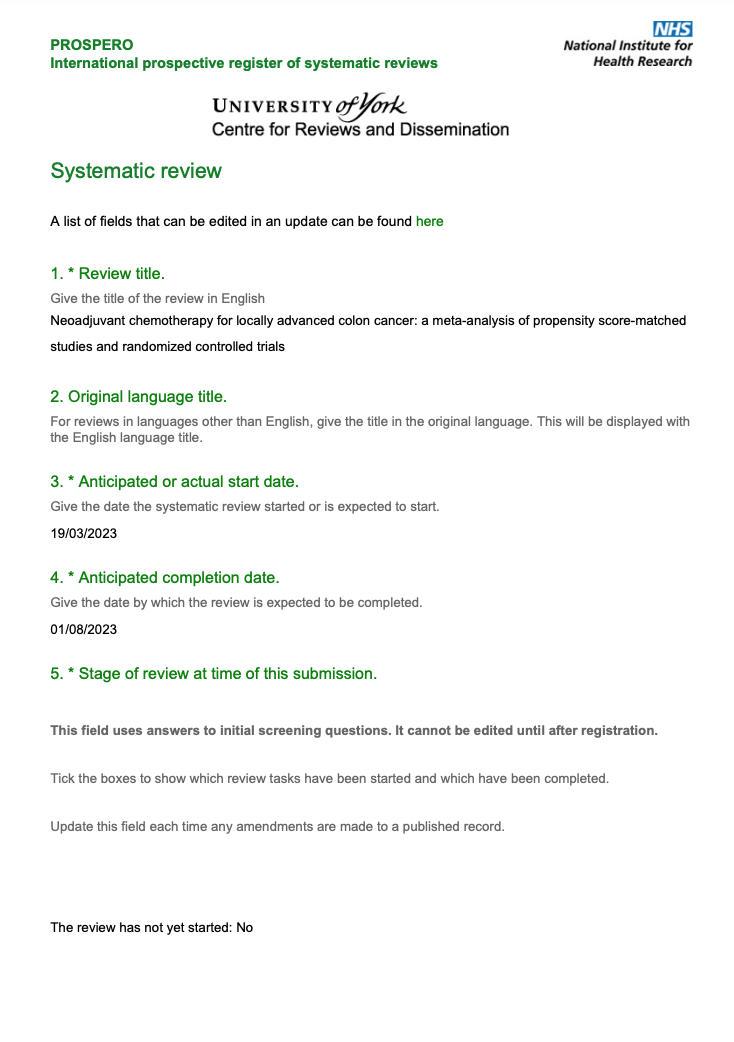


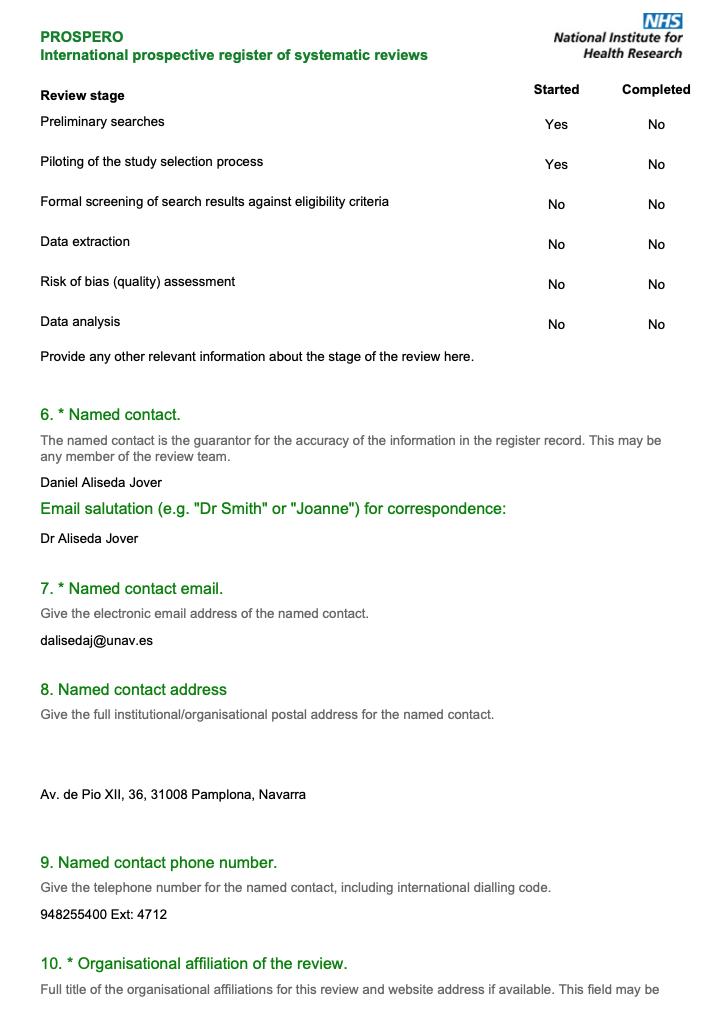


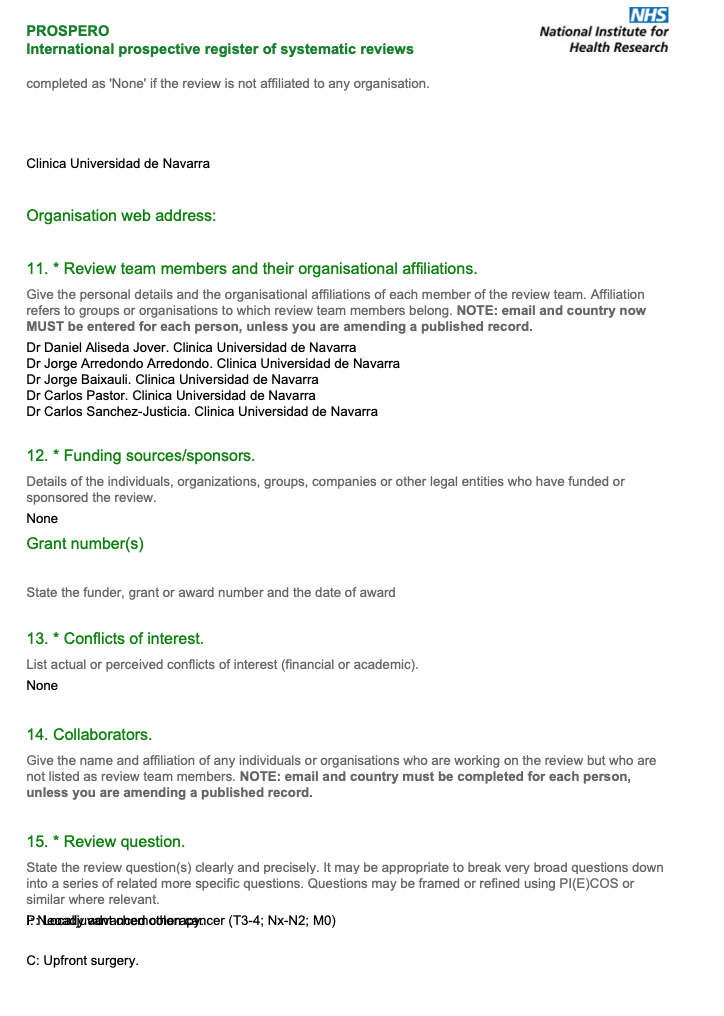


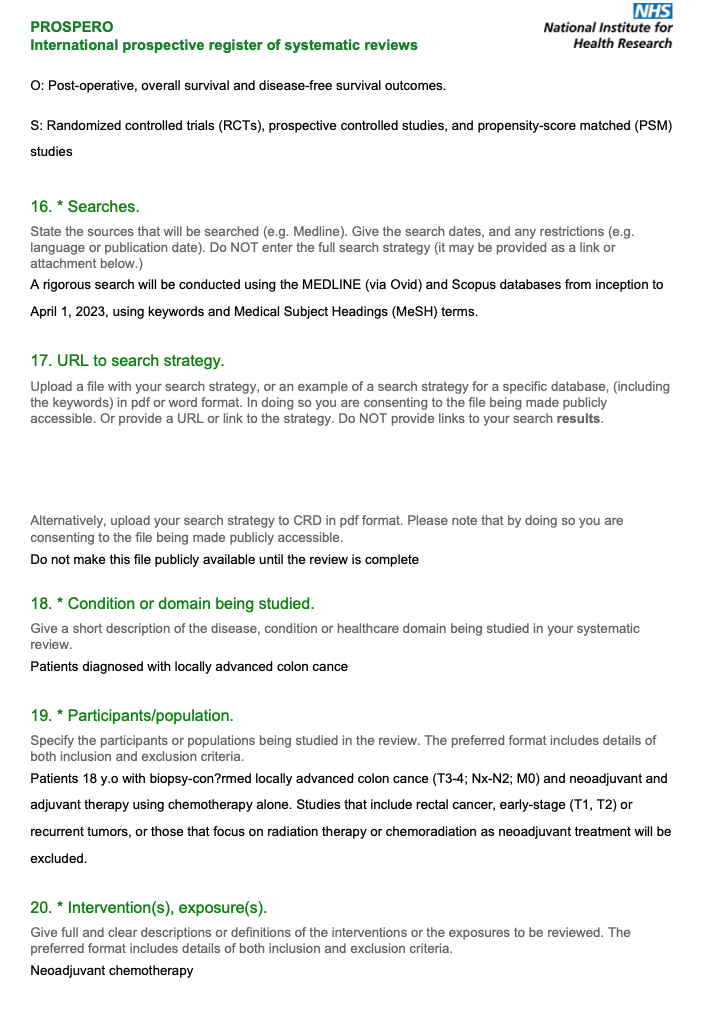


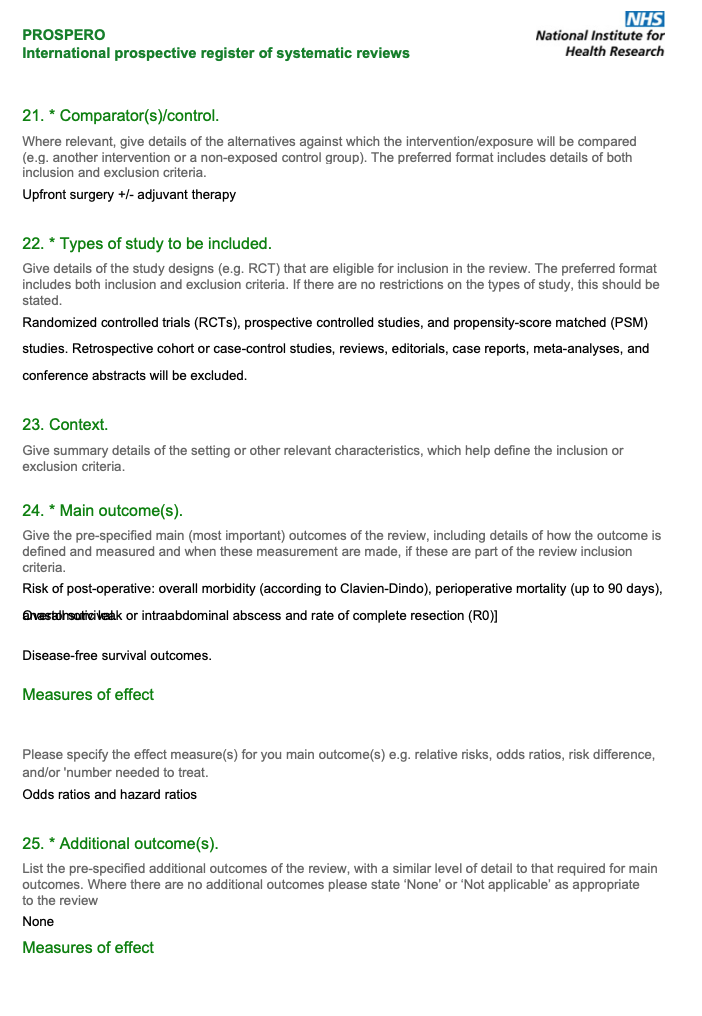


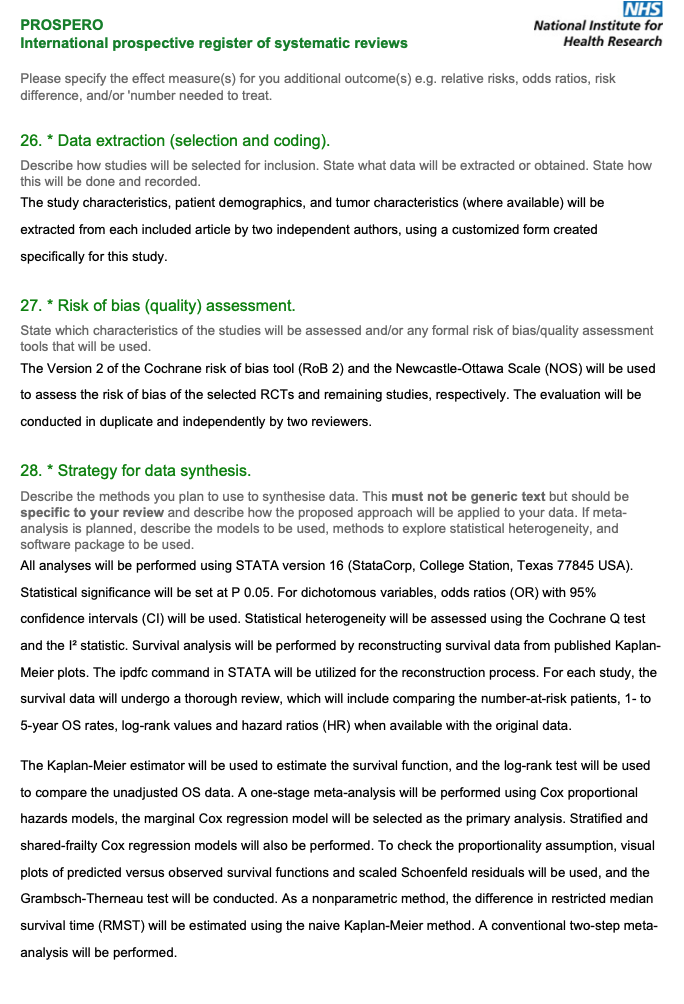


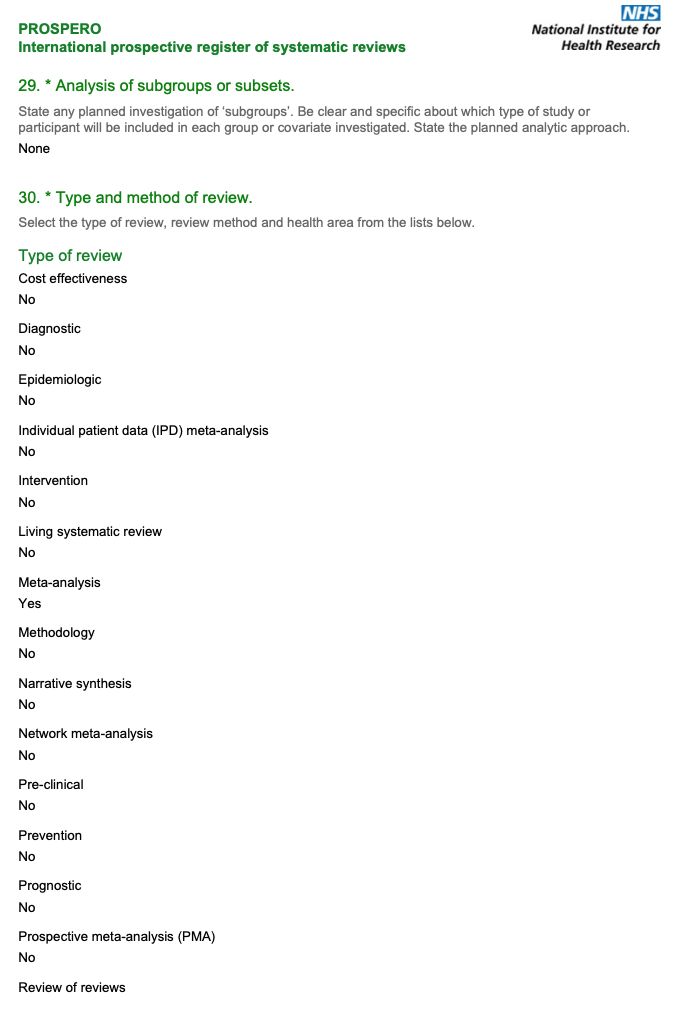


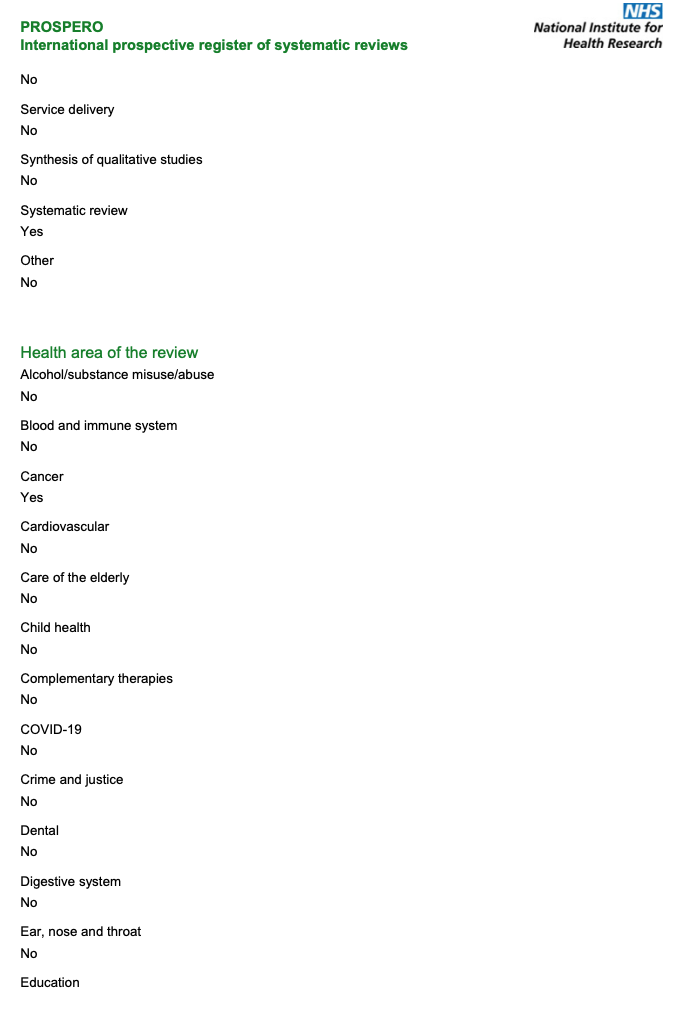


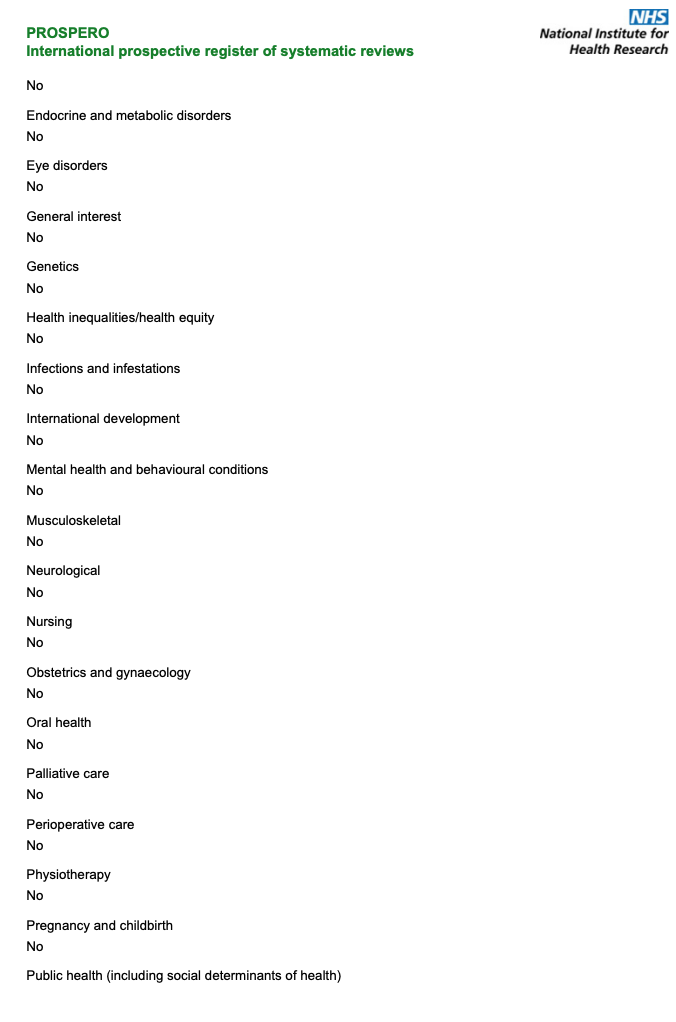


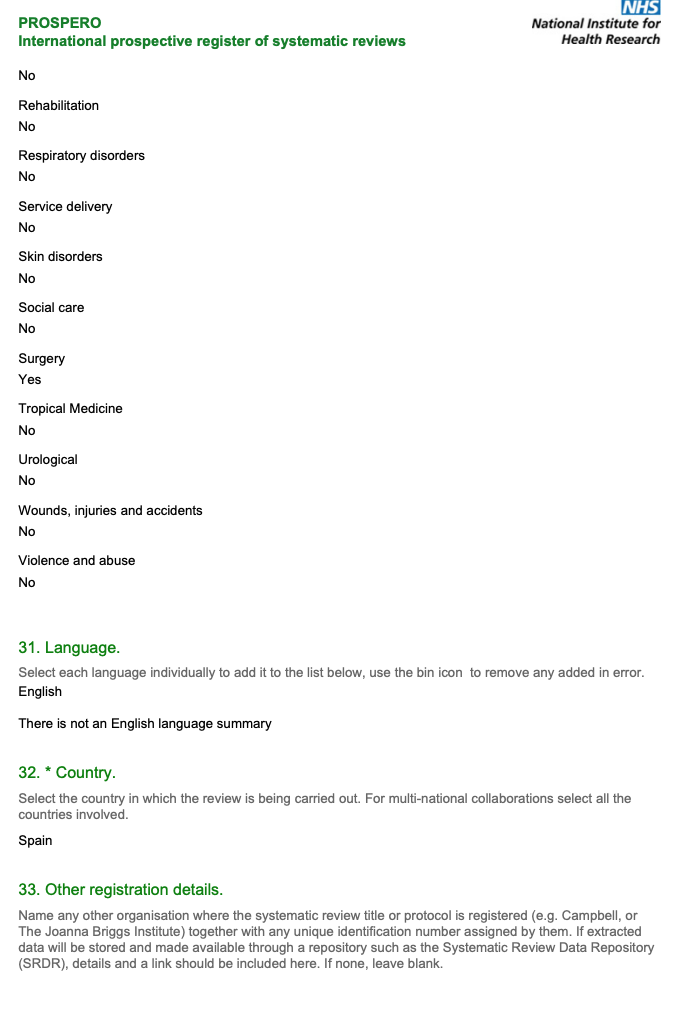


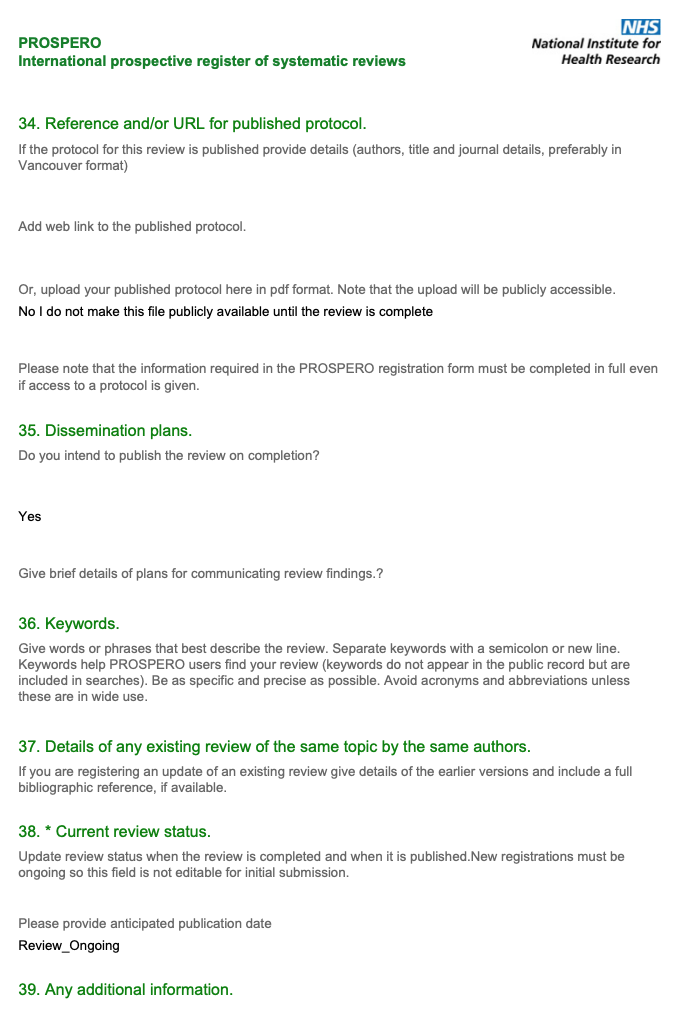


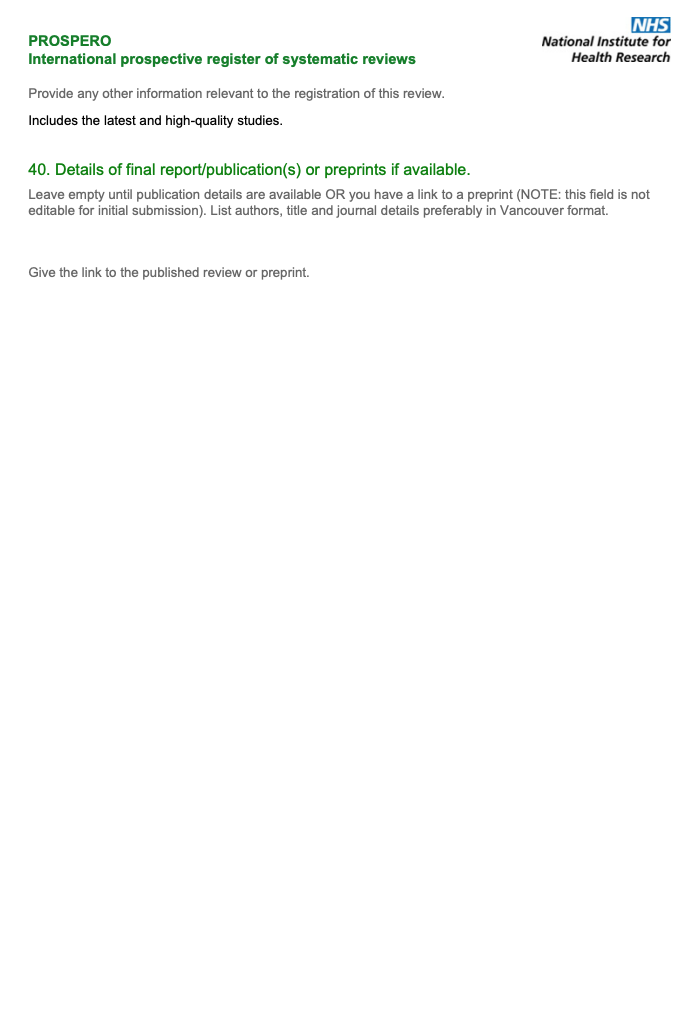


## PRISMA CHECKLIST


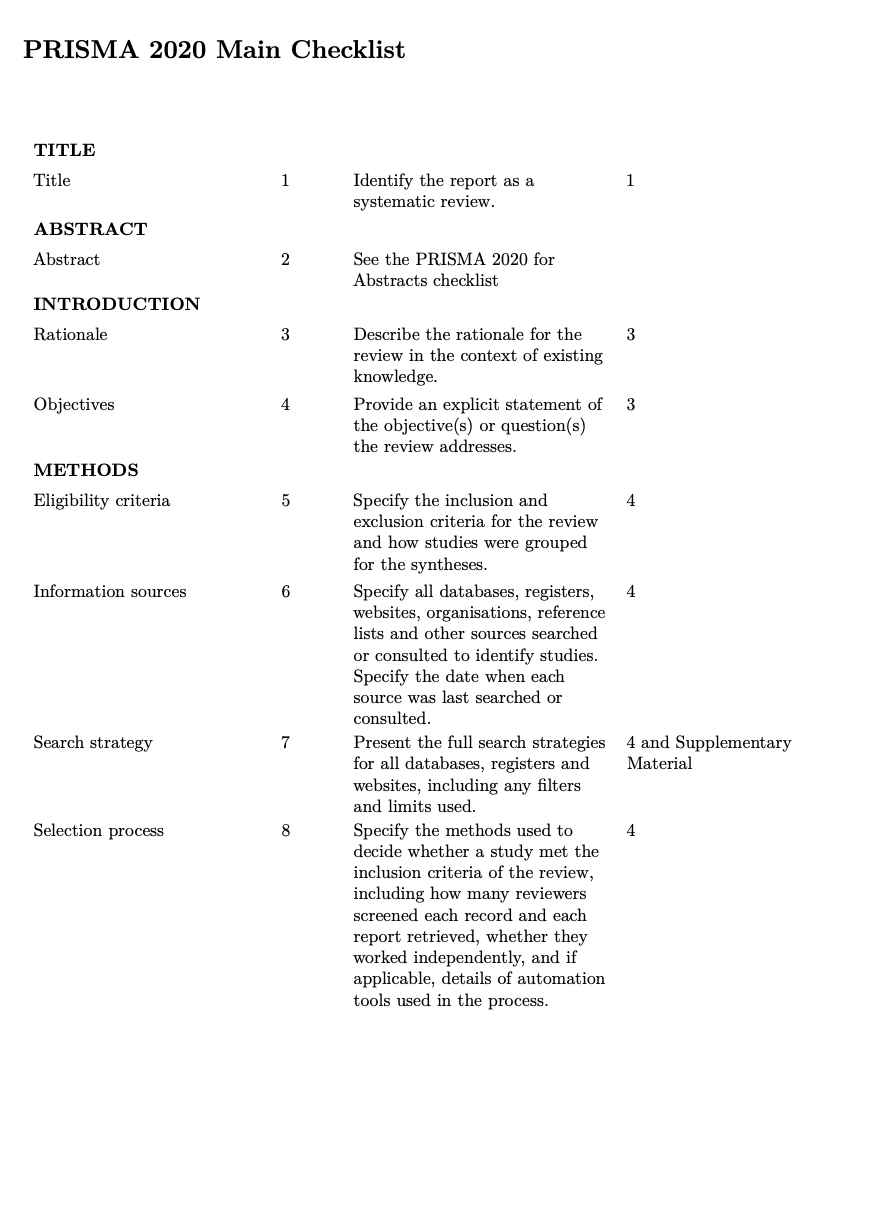


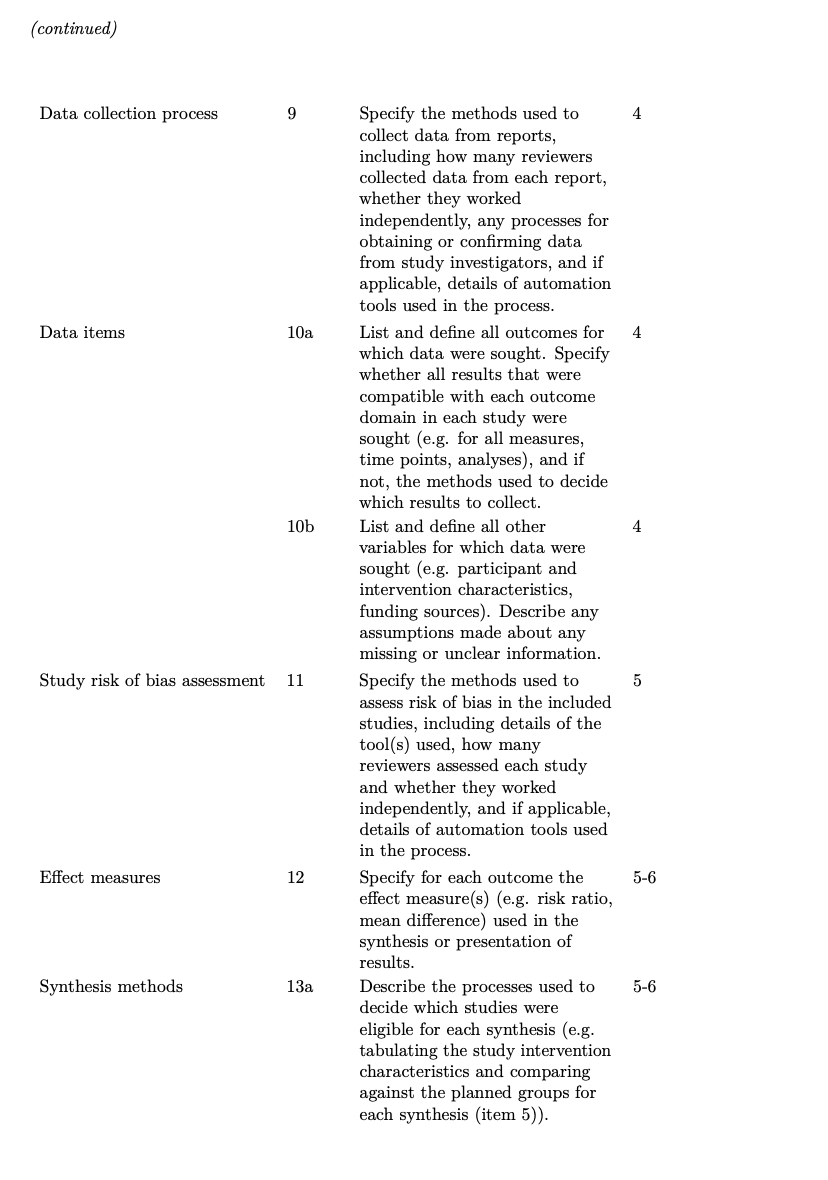


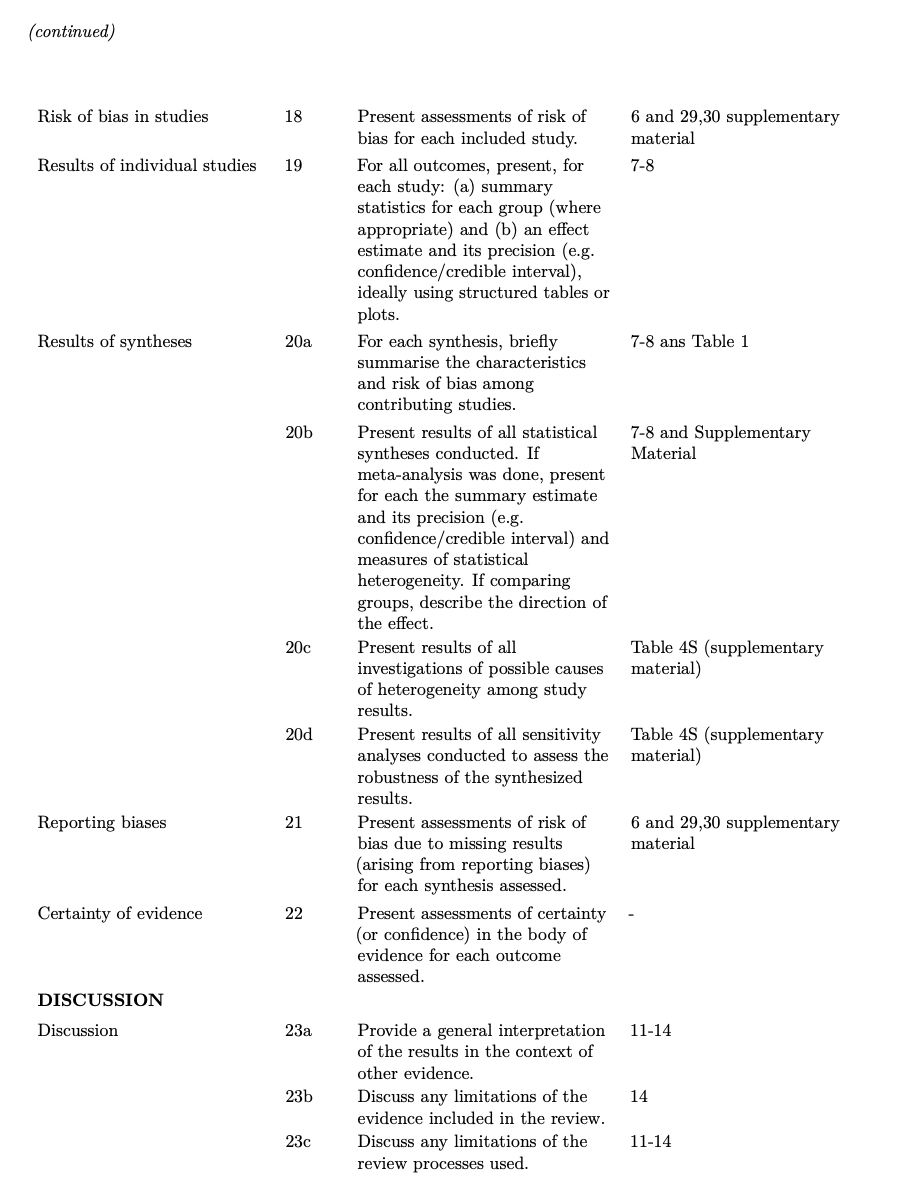


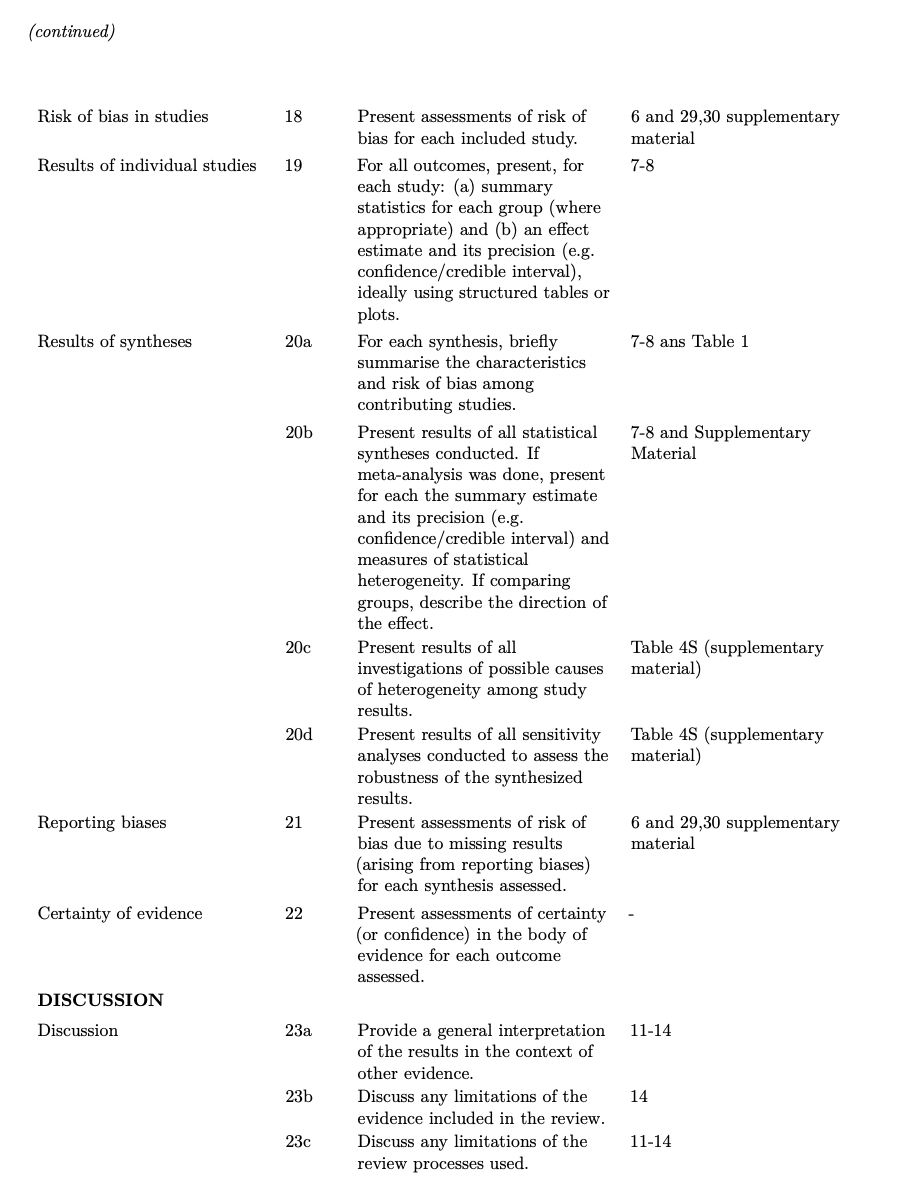


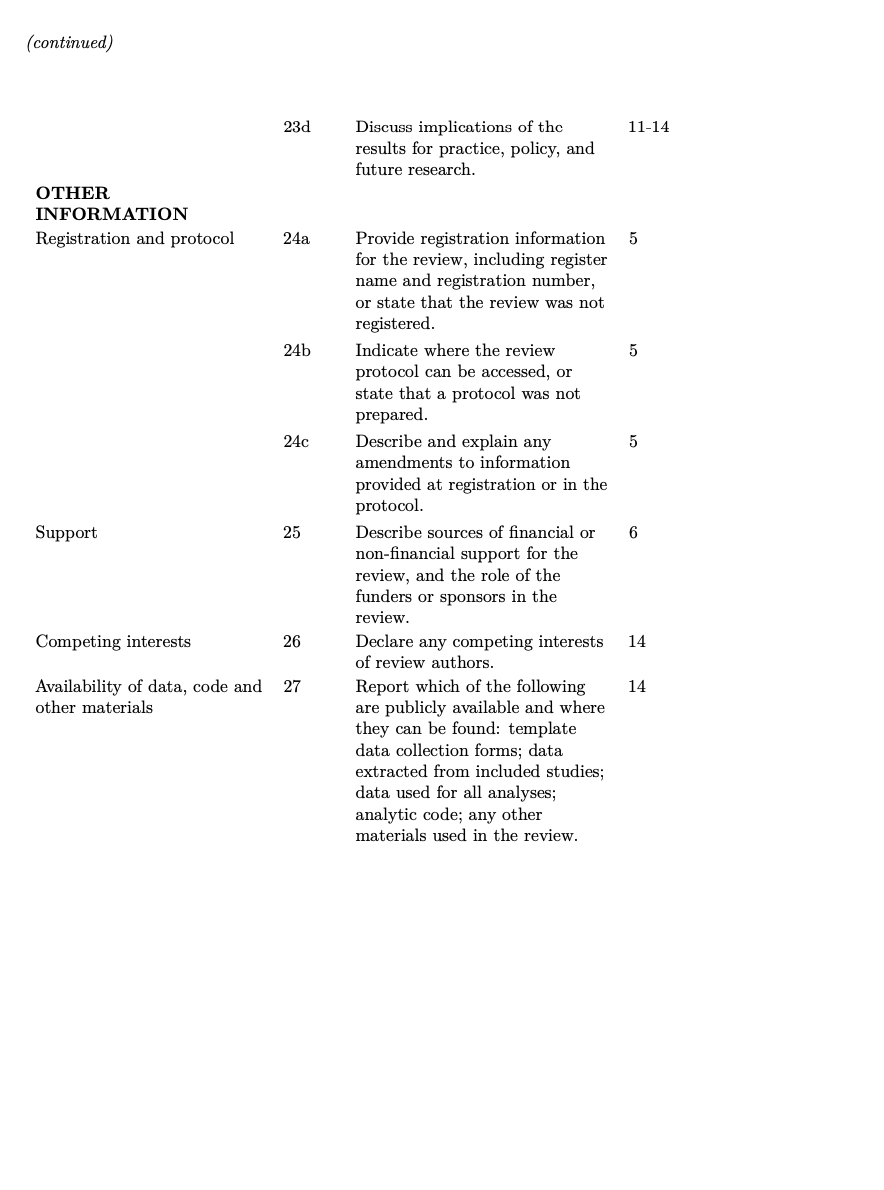


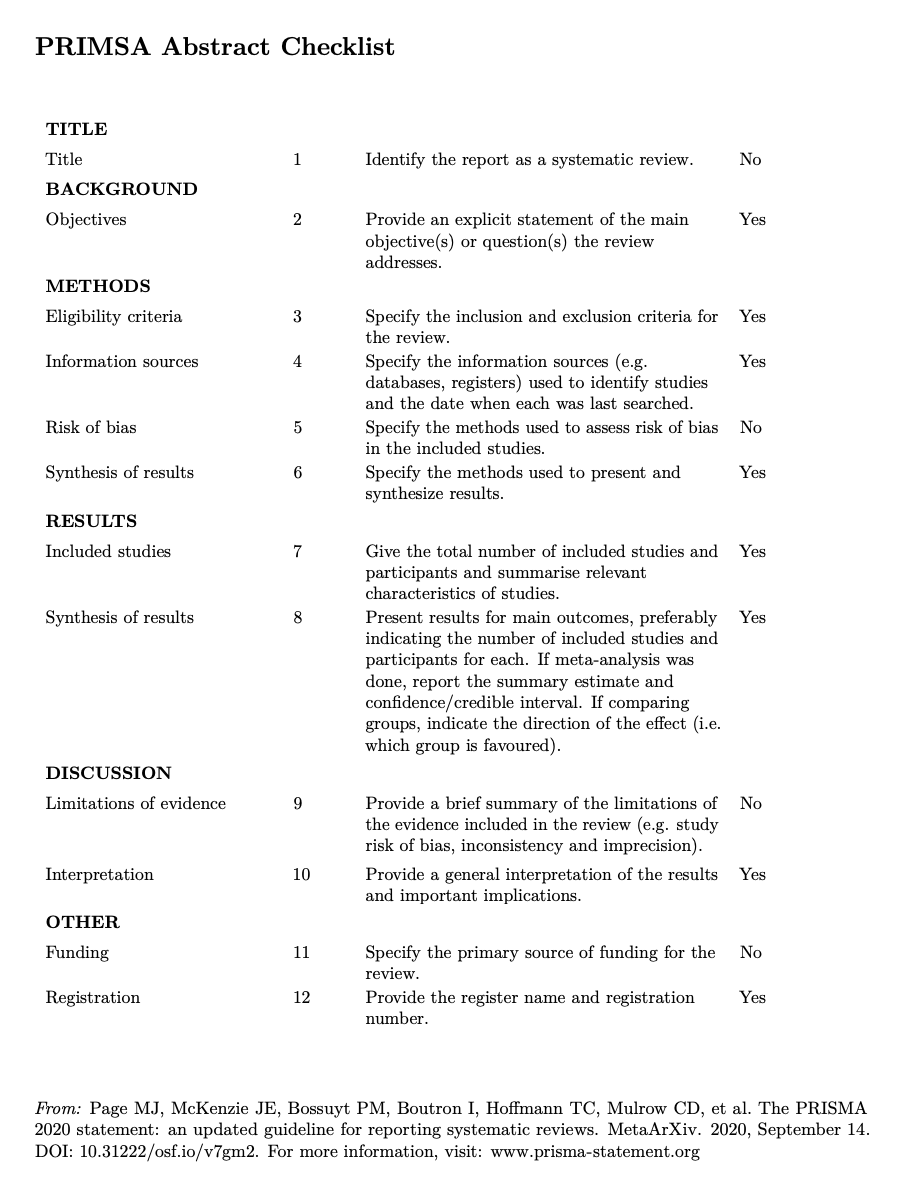


## ADDITIONAL SURVIVAL ANALYSIS

OPTICAL trial (ASCO 2022)^8^

**OVERALL SURVIVAL**

Kaplan–Meier curve from original paper


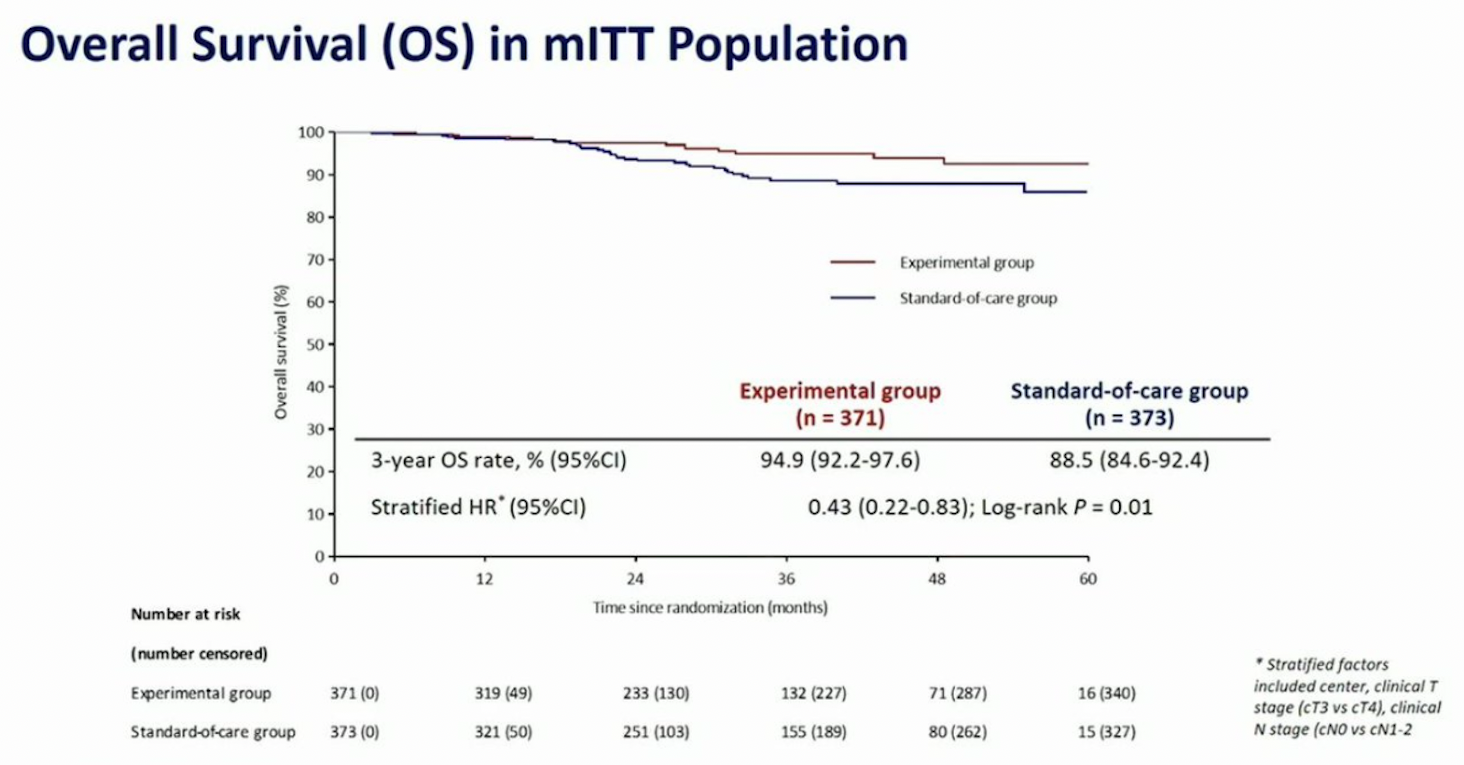


Reconstructed survival curves including number-at-risk tables

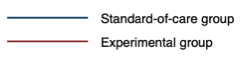

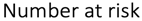


Grambsch–Therneau test


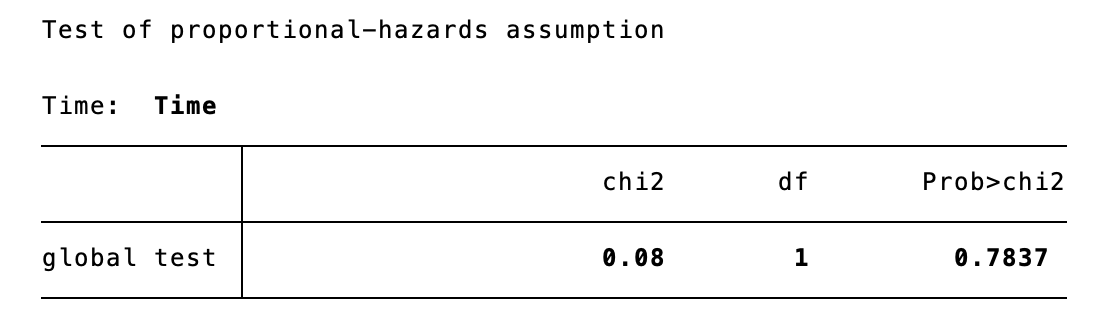
Schoenfeld residuals plot

Predicted versus observed survivor functions

Reconstructed Log-rank test

**
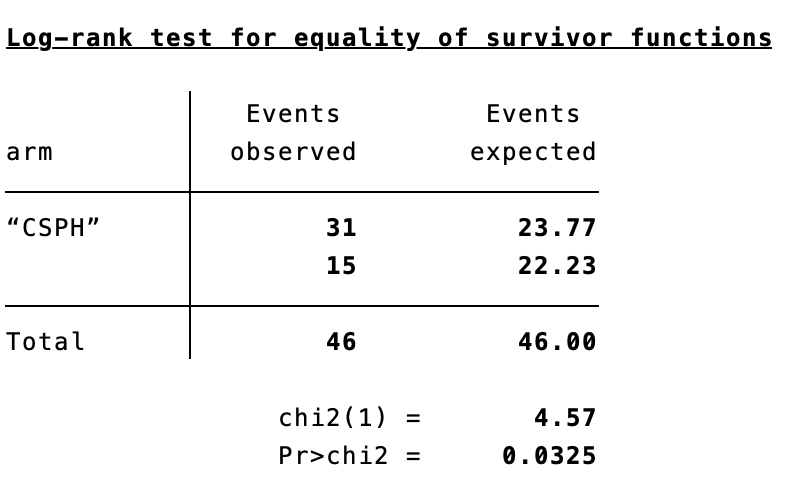
**

Reconstructed 3-year OS rate, % (95% CI)

**Experimental group:** 94.7% (95 CI: 90.8-97.0)

**Standard-of-care group:** 88.3% (95 CI: 83.5-91.8)

**DISEASE FREE SURVIVAL**

Kaplan–Meier curve from original paper


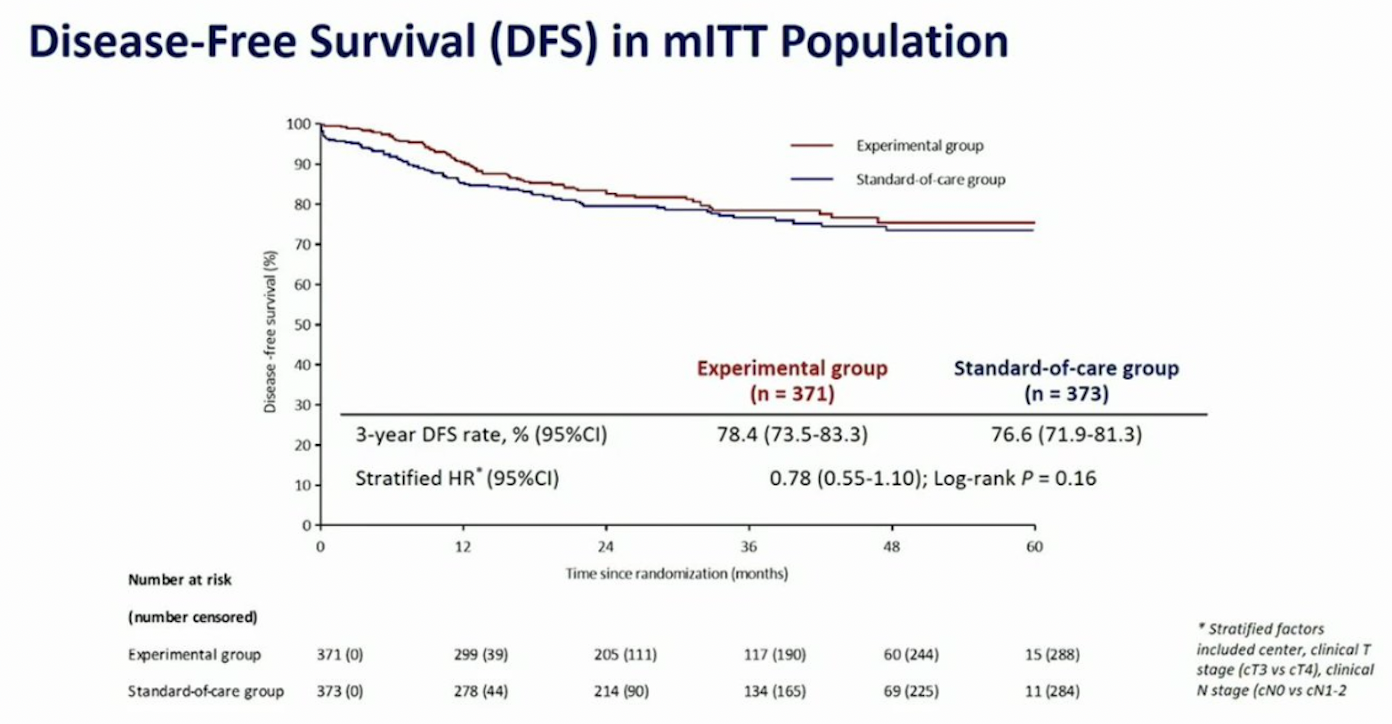


Reconstructed survival curves including number-at-risk tables

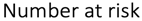


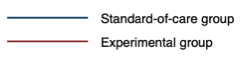


Grambsch–Therneau test


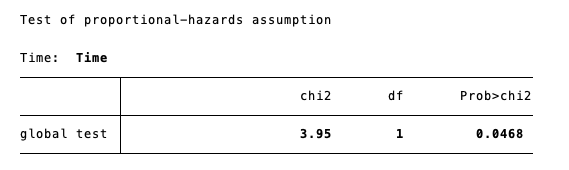


Schoenfeld residuals plot

Predicted versus observed survivor functions

Reconstructed Log-rank test


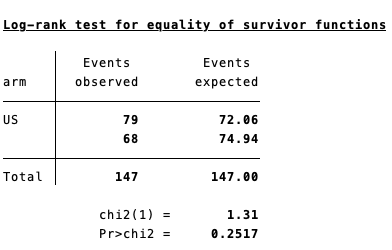


Reconstructed 3-year DFS rate, % (95% CI)

**Experimental group:** 78.4% (95 CI: 73.1-82.8)

**Standard-of-care group:** 76.6% (95 CI: 71.4-81.0)

NeoCol trial (ASCO 2023)^9^

**OVERALL SURVIVAL**

Kaplan–Meier curve from original paper


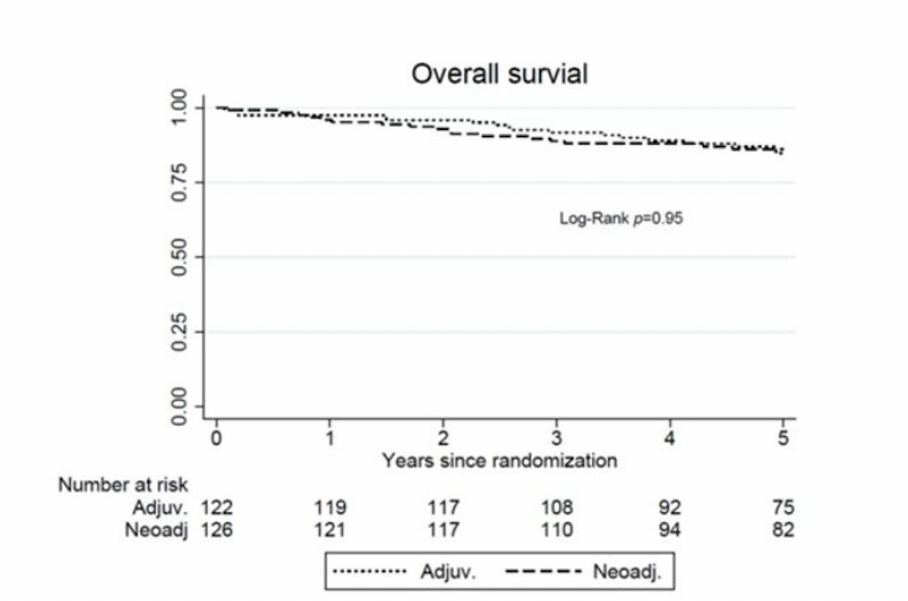


Reconstructed survival curves including number-at-risk tables

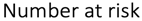


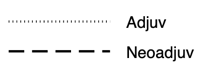


Grambsch–Therneau test


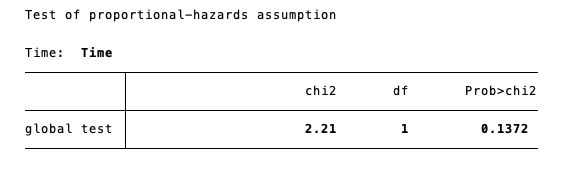


Schoenfeld residuals plot

Predicted versus observed survivor functions

Reconstructed Log-rank test

**
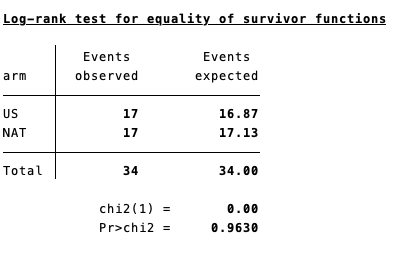
**

**DISEASE FREE SURVIVAL**

Kaplan–Meier curve from original paper


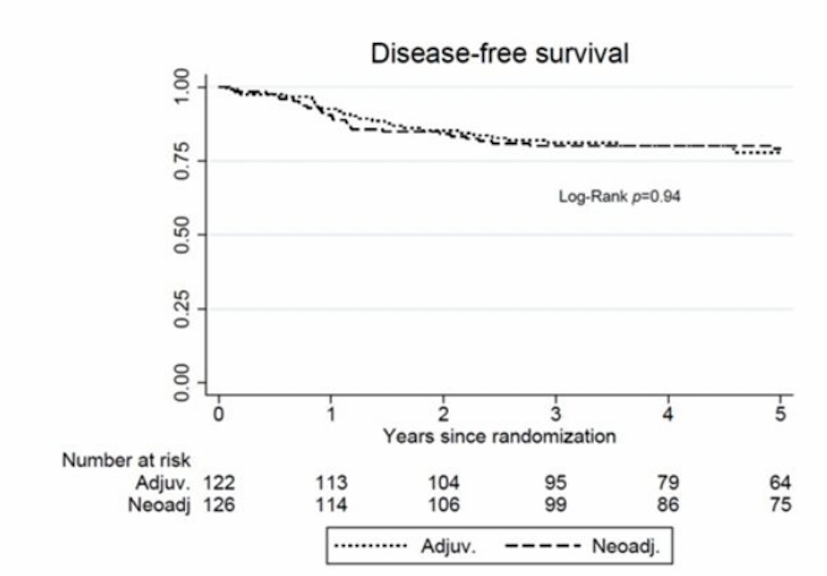


Reconstructed survival curves including number-at-risk tables

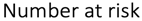


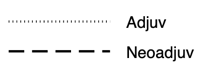


Grambsch–Therneau test


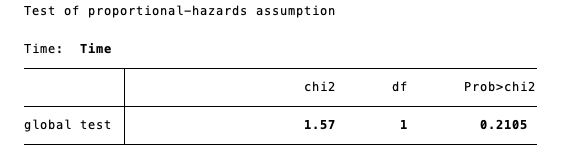


Schoenfeld residuals plot

Predicted versus observed survivor functions

Reconstructed Log-rank test


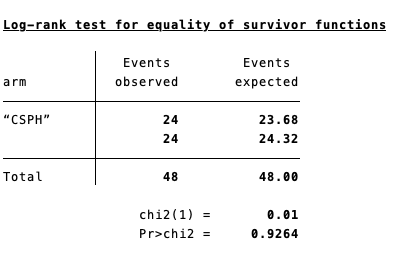


## FINAL ANALYSIS DISEASE FREE-SURVIVAL (including OPTICAL trial)

Reconstructed survival curves including number-at-risk tables


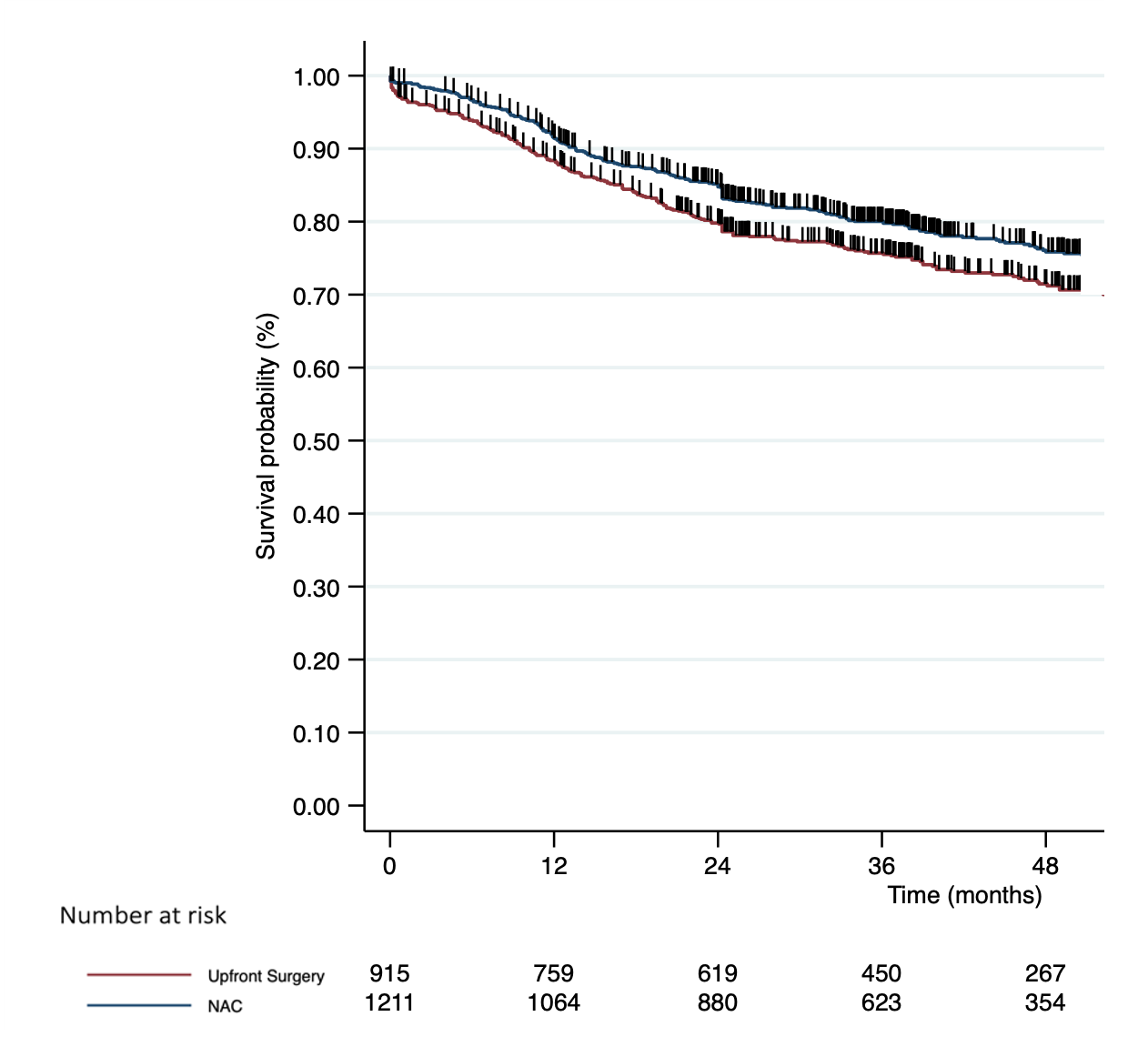


Marginal Cox regression


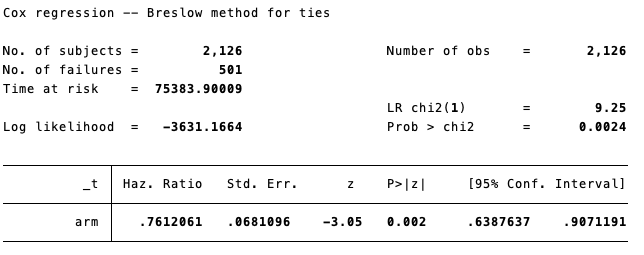


Reconstructed Log-rank test


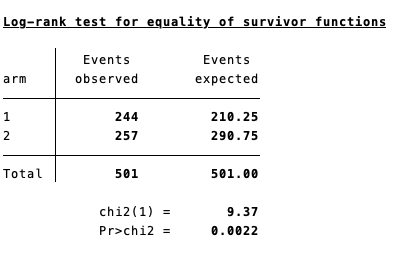


## FINAL ANALYSIS DISEASE FREE-SURVIVAL (including OPTICAL and NeoCol trial)

Reconstructed survival curves including number-at-risk tables


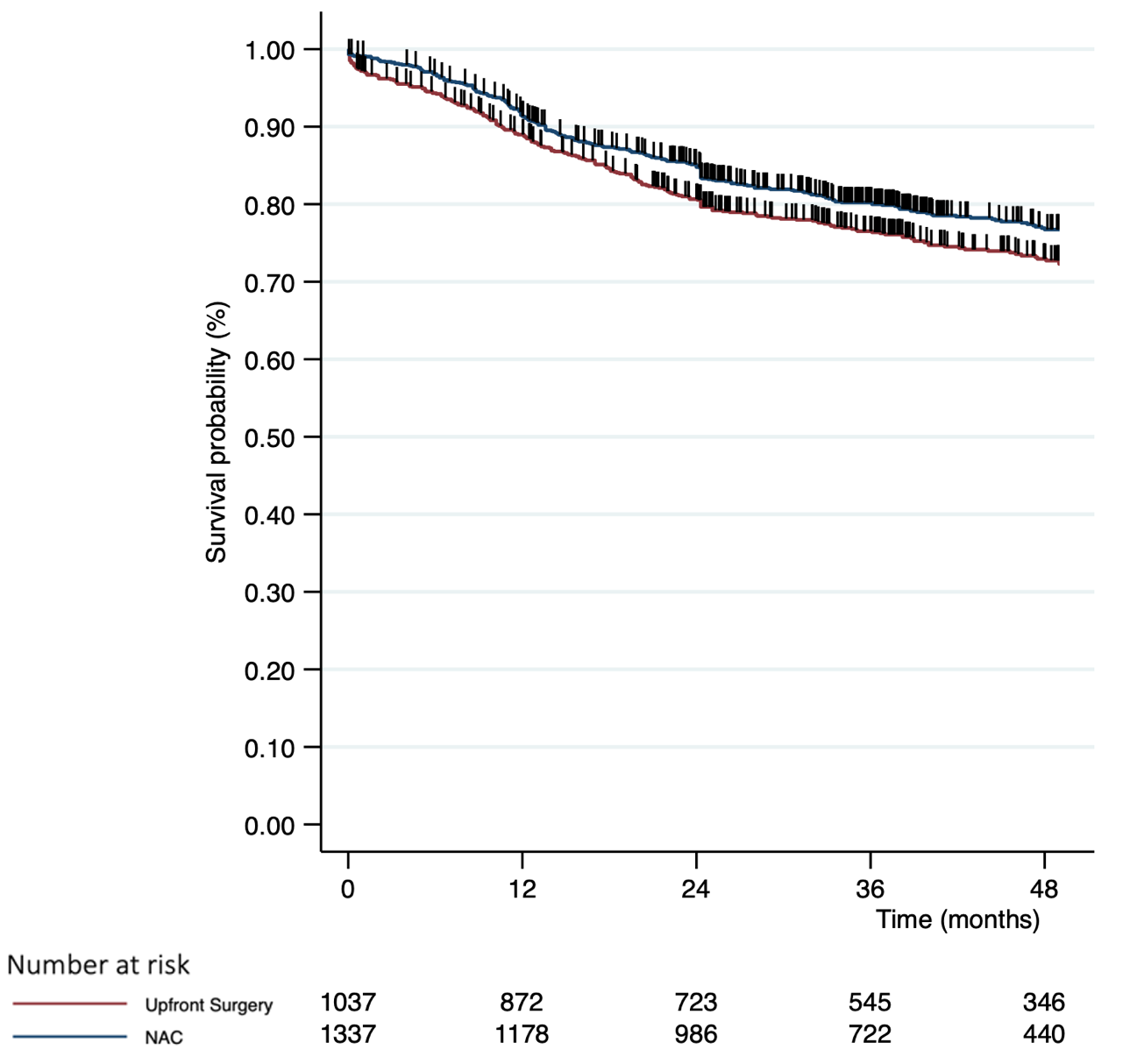


Marginal Cox regression


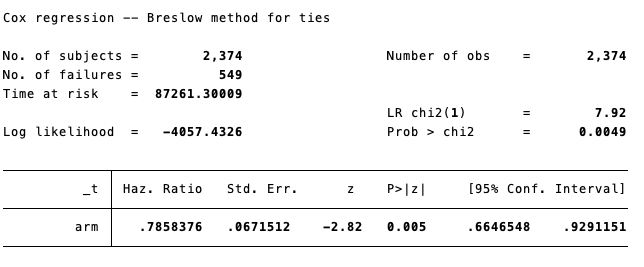


Reconstructed Log-rank test


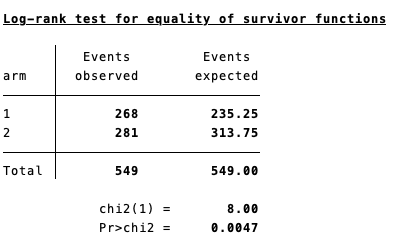


## FINAL ANALYSIS OVERALL SURVIVAL (including NeoCoL trial)

Reconstructed survival curves including number-at-risk tables


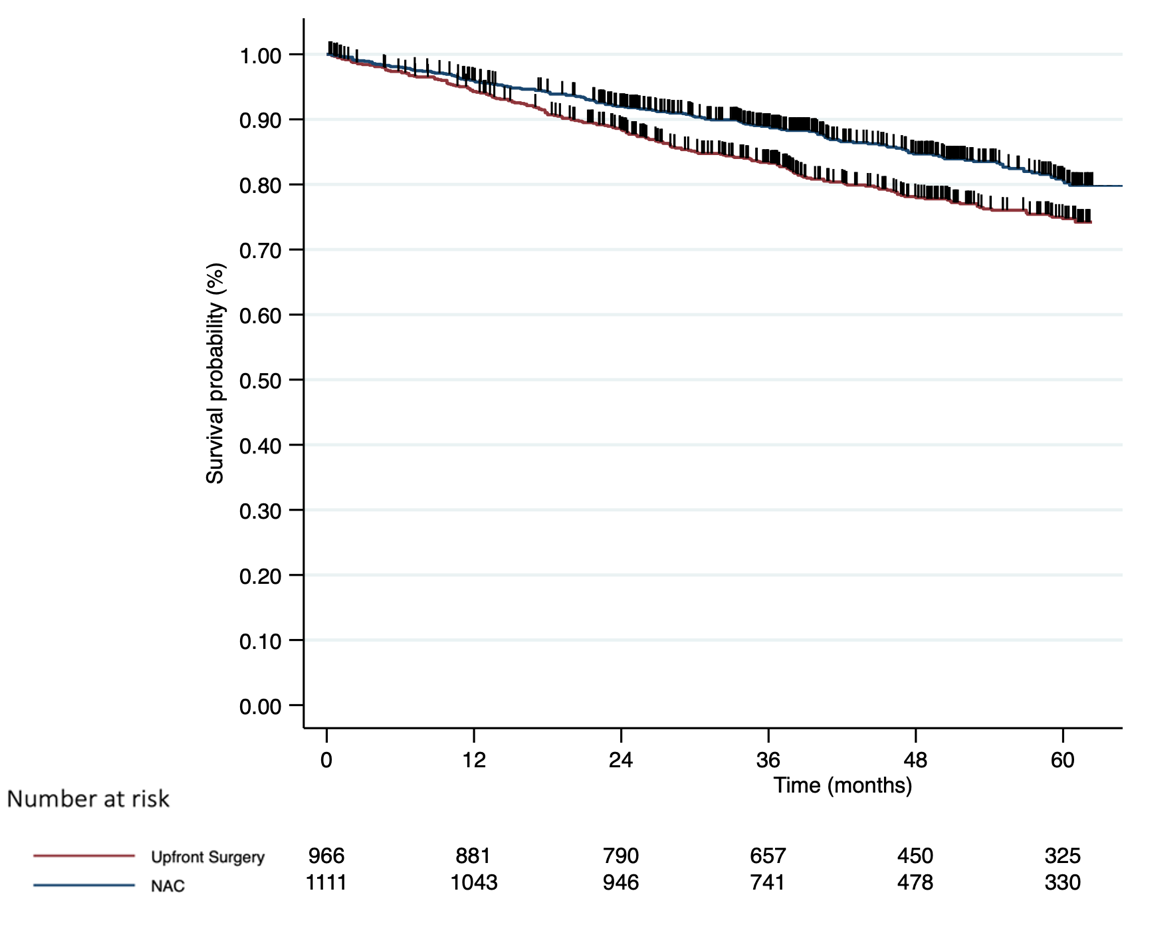


Marginal Cox regression


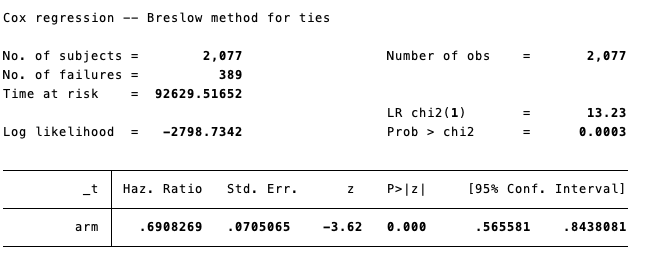


Reconstructed Log-rank test


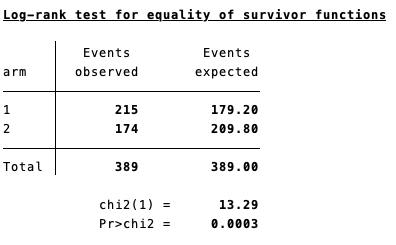


## FINAL ANALYSIS OVERALL SURVIVAL (including NeoCoL and OPTICAL trial)

Reconstructed survival curves including number-at-risk tables


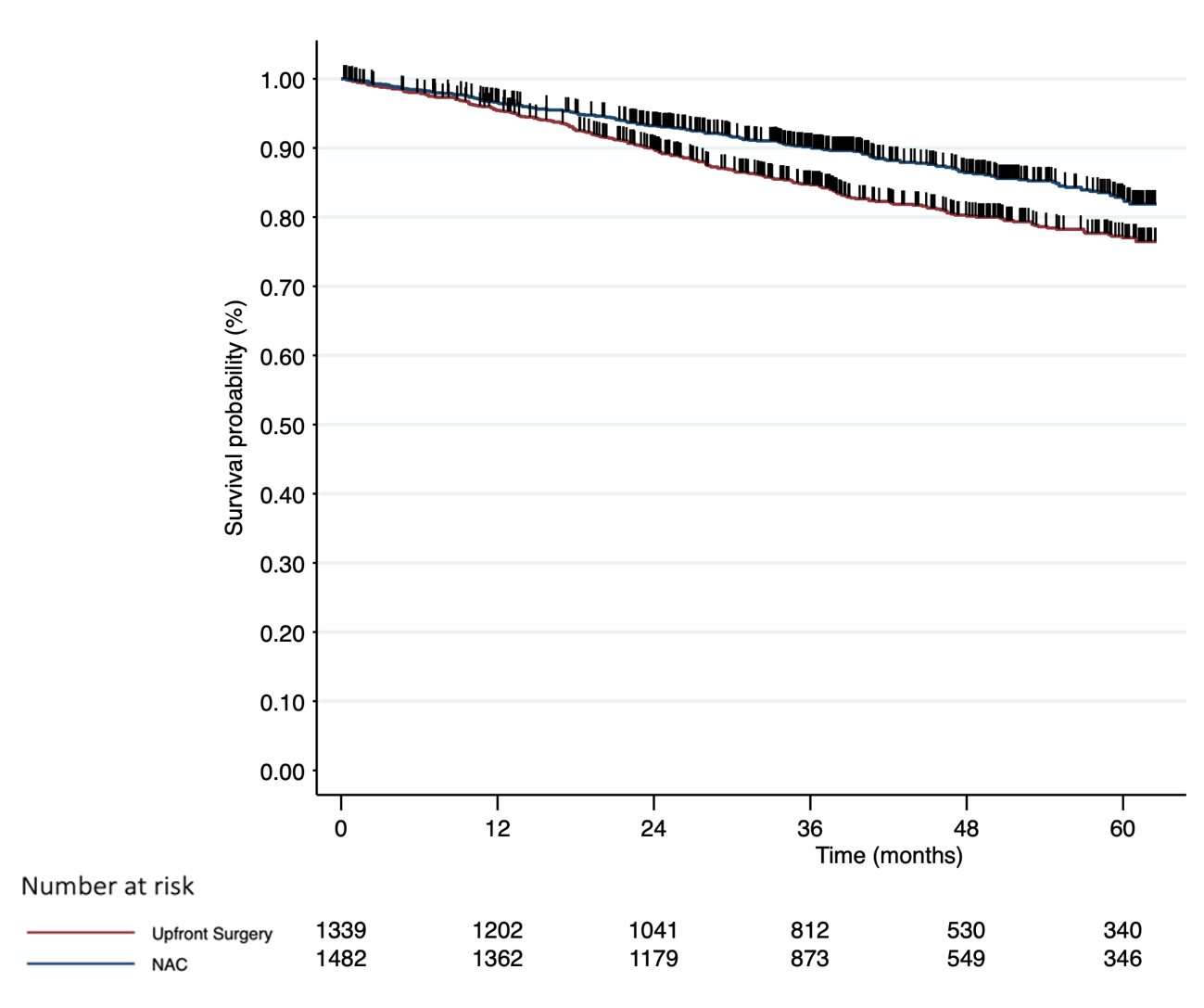


Marginal Cox regression


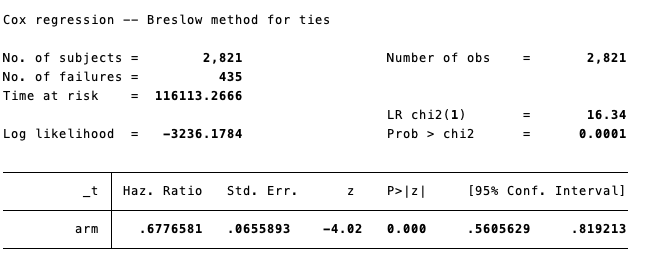


Reconstructed Log-rank test


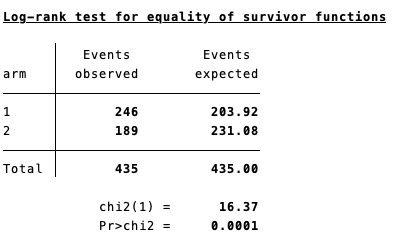


# REFERENCES

1 Morton D, Seymour M, Magill L, Handley K, Glasbey J, Glimelius B, *et al.* Preoperative Chemotherapy for Operable Colon Cancer: Mature Results of an International Randomized Controlled Trial. *J Clin Oncol*. 2023; JCO2200046.

2 Zeng W, Liu Y, Wang C, Yang C, Lin S, Li W. Efficacy and Safety of Neoadjuvant Chemotherapy Combined with Adjuvant Chemotherapy for Locally Advanced Colon Cancer: A Propensity Score-Matching Analysis. *Medicina*. 2022; **58**: 1505.

3 Han JG, Wang ZJ, Dai Y, Li XR, Qian Q, Wang GY, *et al.* Short-term Outcomes of Elective Surgery Following Self-Expandable Metallic Stent and Neoadjuvant Chemotherapy in Patients With Left-Sided Colon Cancer Obstruction. *Dis Colon Rectum*. 2022; **Publish Ahead of Print**.

4 Karoui M, Gallois C, Piessen G, Legoux J, Barbier E, Chaisemartin CD, *et al.* Does neoadjuvant FOLFOX chemotherapy improve the prognosis of high‐risk Stage II and III colon cancers? Three years’ follow‐up results of the PRODIGE 22 phase II randomized multicentre trial. *Colorectal Dis*. 2021; **23**: 1357–1369.

5 Gooyer J-M de, Verstegen MG, Lam-Boer J ’t, Radema SA, Verhoeven RHA, Verhoef C, *et al.* Neoadjuvant Chemotherapy for Locally Advanced T4 Colon Cancer: A Nationwide Propensity-Score Matched Cohort Analysis. *Digest Surg*. 2020; **37**: 292–301.

6 Laursen M, Dohrn N, Gögenur I, Klein MF. Neoadjuvant chemotherapy in patients undergoing colonic resection for locally advanced nonmetastatic colon cancer: A nationwide propensity score matched cohort study. *Colorectal Dis*. 2022; **24**: 954–964.

7 Karoui M, Rullier A, Piessen G, Legoux JL, Barbier E, Chaisemartin CD, *et al.* Perioperative FOLFOX 4 Versus FOLFOX 4 Plus Cetuximab Versus Immediate Surgery for High-Risk Stage II and III Colon Cancers: A Phase II Multicenter Randomized Controlled Trial (PRODIGE 22). *Ann Surg*. 2020; **271**: 637–645.

8 Perioperative chemotherapy with mFOLFOX6 or CAPOX for patients with locally advanced colon cancer (OPTICAL)- A multicenter, randomized, phase 3 trial..pdf. *Journal of Clinical Oncology*. 2022 Jun 2; **40**.

9 Jensen LH, Kjaer ML, Larsen FO, Hollander NH, Rahr HB, Pfeffer F, *et al.* Phase III randomized clinical trial comparing the efficacy of neoadjuvant chemotherapy and standard treatment in patients with locally advanced colon cancer: The NeoCol trial. *J Clin Oncol*. 2023; **41**: LBA3503–LBA3503.
